# Pharmacological hallmarks of allostery at the M4 muscarinic receptor elucidated through structure and dynamics

**DOI:** 10.7554/eLife.83477

**Published:** 2023-05-30

**Authors:** Ziva Vuckovic, Jinan Wang, Vi Pham, Jesse I Mobbs, Matthew J Belousoff, Apurba Bhattarai, Wessel AC Burger, Geoff Thompson, Mahmuda Yeasmin, Vindhya Nawaratne, Katie Leach, Emma T van der Westhuizen, Elham Khajehali, Yi-Lynn Liang, Alisa Glukhova, Denise Wootten, Craig W Lindsley, Andrew Tobin, Patrick Sexton, Radostin Danev, Celine Valant, Yinglong Miao, Arthur Christopoulos, David M Thal

**Affiliations:** 1 Drug Discovery Biology, Monash Institute of Pharmaceutical Sciences, Monash University Parkville Australia; 2 Center for Computational Biology and Department of Molecular Biosciences, University of Kansas Lawrence United States; 3 ARC Centre for Cryo-electron Microscopy of Membrane Proteins, Monash Institute of Pharmaceutical Sciences, Monash University Parkville Australia; 4 Department of Pharmacology, Warren Center for Neuroscience Drug Discovery and Department of Chemistry, Warren Center for Neuroscience Drug Discovery, Vanderbilt University Nashville United States; 5 The Centre for Translational Pharmacology, Advanced Research Centre (ARC), College of Medical, Veterinary and Life Sciences, University of Glasgow Glasgow United Kingdom; 6 Graduate School of Medicine, University of Tokyo Tokyo Japan; 7 Neuromedicines Discovery Centre, Monash University Parkville Australia; Duke University Medical Center United States; National Institute of Neurological Disorders and Stroke, National Institutes of Health United States

**Keywords:** allostery, drug discovery, molecular dynamics, molecular pharmacology, muscarinic acetylcholine receptors, structural biology, None

## Abstract

Allosteric modulation of G protein-coupled receptors (GPCRs) is a major paradigm in drug discovery. Despite decades of research, a molecular-level understanding of the general principles that govern the myriad pharmacological effects exerted by GPCR allosteric modulators remains limited. The M_4_ muscarinic acetylcholine receptor (M_4_ mAChR) is a validated and clinically relevant allosteric drug target for several major psychiatric and cognitive disorders. In this study, we rigorously quantified the affinity, efficacy, and magnitude of modulation of two different positive allosteric modulators, LY2033298 (LY298) and VU0467154 (VU154), combined with the endogenous agonist acetylcholine (ACh) or the high-affinity agonist iperoxo (Ipx), at the human M_4_ mAChR. By determining the cryo-electron microscopy structures of the M_4_ mAChR, bound to a cognate G_i1_ protein and in complex with ACh, Ipx, LY298-Ipx, and VU154-Ipx, and applying molecular dynamics simulations, we determine key molecular mechanisms underlying allosteric pharmacology. In addition to delineating the contribution of spatially distinct binding sites on observed pharmacology, our findings also revealed a vital role for orthosteric and allosteric ligand–receptor–transducer complex stability, mediated by conformational dynamics between these sites, in the ultimate determination of affinity, efficacy, cooperativity, probe dependence, and species variability. There results provide a holistic framework for further GPCR mechanistic studies and can aid in the discovery and design of future allosteric drugs.

## Introduction

**Figure 1.**
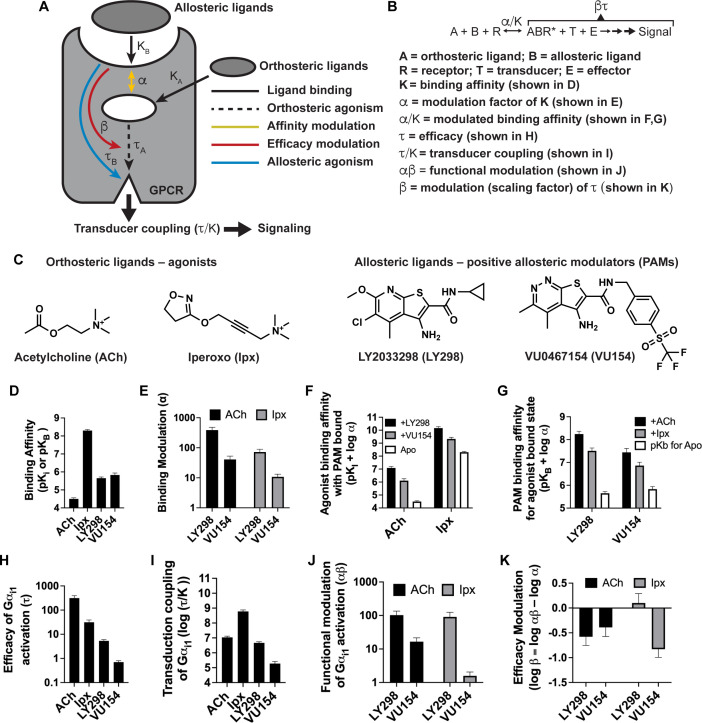
Pharmacological characterization of the positive allosteric modulators (PAMs), LY298 and VU154, with acetylcholine (ACh) and iperoxo (Ipx) at the human M_4_ muscarinic acetylcholine receptor (mAChR). (**A**) Schematic of the pharmacological parameters that define effects of orthosteric and allosteric ligands on a G protein-coupled receptor (GPCR). (**B**) A simplified schematic diagram of the Black–Leff operational model to quantify agonism, allosteric modulation, and agonist bias with pharmacological parameters defined (Black and Leff, 1983). (**C**) 2D chemical structures of the orthosteric and allosteric ligands used in this study. (**D–G**) Key pharmacological parameters for interactions between orthosteric and allosteric ligands in [^3^H]-N-methylscopolamine ([^3^H]-NMS) binding assays. (**D**) Equilibrium binding affinities (pK_i_ and pK_B_) and (**E**) the degree of binding modulation (α) between the agonists and PAMs resulting in the modified binding affinities (**F**) α/K_A_ and (**G**) α/K_B_. (**H–K**) Key pharmacological parameters relating to Gα_i1_ activation for interactions between orthosteric and allosteric ligands measured with the TruPath assay (Figure 1—figure supplement 1). (**H**) The signaling efficacy (τ_A_ and τ_B_) and (**I**) transduction coupling coefficients (log (τ/K)) of each ligand. (**J**) The functional cooperativity (αβ) between ligands and (**K**) the efficacy modulation (β) between ligands. All data are mean ± SEM of three or more independent experiments performed in duplicate or triplicate with the pharmacological parameters determined using a global fit of the data. The error in (**F, G, K**) was propagated using the square root of the sum of the squares. See Table 1. Concentration–response curves are shown in Figure 1—figure supplement 1. Figure 1—source data 1.Related to Figure 1D–K.

Over the past 40 y, there have been major advances to the analytical methods that allow for the quantitative determination of the pharmacological parameters that characterize G protein-coupled receptor (GPCR) signaling and allosteric modulation (Figure 1A and B). These analytical methods are based on the operational model of agonism (Black and Leff, 1983) and have been extended or modified to account for allosteric modulation (Leach et al., 2007), biased agonism (Kenakin, 2012), and even biased allosteric modulation (Slosky et al., 2021). Collectively, these models and subsequent key parameters (Figure 1B) are used to guide allosteric drug screening, selectivity, efficacy, and ultimately, clinical utility, and provide the foundation for modern GPCR drug discovery (Wootten et al., 2013). Yet, a systematic understanding of how these pharmacological parameters relate to the molecular structure and dynamics of GPCRs remains elusive.

**Figure 1—figure supplement 1.**
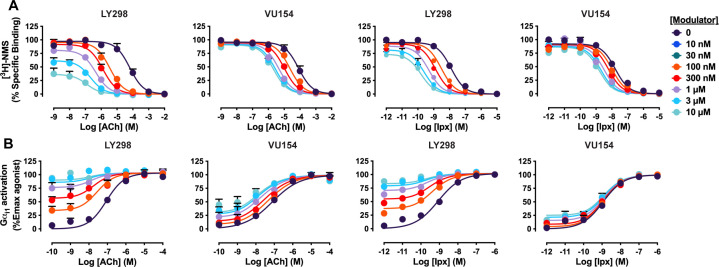
Concentration–response curves of interactions between the orthosteric ligands (acetylcholine [ACh], iperoxo [Ipx]) and the allosteric ligands (LY298, VU154) at the human M_4_ muscarinic acetylcholine receptor (mAChR). (**A**) [^3^H]-N-methylscopolamine ([^3^H]-NMS) binding assays. (**B**) Gα_i1_ activation using the TruPath assay. All data points are mean ± SEM of three or more independent experiments performed in duplicate or triplicate with the pharmacological parameters determined from a global fit of the data. Parameters quantifying the data are shown in Figure 1 and Table 1. Figure 1—figure supplement 1—source data 1.Related to Figure 1—figure supplement 1.

**Figure 1—figure supplement 2.**
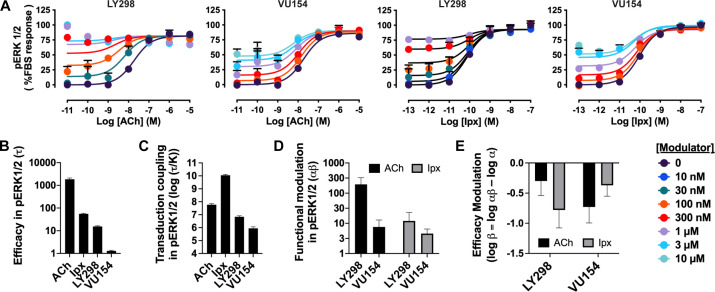
Pharmacological characterization of the positive allosteric modulators (PAMs), LY298 and VU154, with acetylcholine (ACh) and iperoxo (Ipx) in pERK1/2 signaling assays. (**A**) Concentration–response curves of interactions between the orthosteric and allosteric ligands at the human M_4_ mAChR in the pERK1/2 signaling assay. (**B–E**) Quantification of data from (**A**) to calculate (**B**) the signaling efficacy (τ_A_ and τ_B_), (**C**) the transduction coupling coefficients (log (τ/K)) of each ligand, (**D**) the functional cooperativity (αβ) between ligands, and (**E**) the efficacy modulation (β) between ligands. All data are mean ± SEM of three or more independent experiments performed in duplicate or triplicate with the pharmacological parameters determined from a global fit of the data. The error in (**E**) was propagated using the square root of the sum of the squares. Pharmacological parameters are reported in Table 1. Figure 1—figure supplement 2—source data 1.Related to Figure 1—figure supplement 2.

The muscarinic acetylcholine receptors (mAChRs) are an important family of five Class A GPCRs that have long served as model systems for understanding GPCR allostery (Conn et al., 2009). The mAChRs have been notoriously difficult to exploit therapeutically and selectively due to high-sequence conservation within their orthosteric binding domains (Burger et al., 2018). However, the discovery of highly selective positive allosteric modulators (PAMs) for some mAChR subtypes has paved the way for novel approaches to exploit these high-value drug targets (Chan et al., 2008; Gentry et al., 2014; Marlo et al., 2009). X-ray crystallography and cryo-electron microscopy (cryo-EM) have been used to determine inactive state structures for all five mAChR subtypes (Haga et al., 2012; Kruse et al., 2012; Thal et al., 2016; Vuckovic et al., 2019) and active state structures of the M_1_ and M_2_ mAChRs (Maeda et al., 2019). For the M_2_ mAChR, this includes structures co-bound with the high-affinity agonist iperoxo (Ipx) and the PAM LY2119620 in complex with a G protein mimetic nanobody (Kruse et al., 2013) and the transducers G_o_ (Maeda et al., 2019) and β-arrestin1 (Staus et al., 2020). These M_2_ mAChR structures were foundational to validating the canonical mAChR allosteric site but are limited to only one agonist (iperoxo) and one PAM (LY2119620) and do not account for the vast pharmacological properties of ligands targeting mAChRs. A recent nuclear magnetic resonance (NMR) study of the M_2_ mAChR revealed differences in the conformational landscape of the M_2_ mAChR when bound to different agonists, but no clear link was established between the properties of the ligands and the conformational states of the receptor (Xu et al., 2019). The M_4_ mAChR subtype is of major therapeutic interest due to its expression in regions of the brain that are rich in dopamine and dopamine receptors, where it regulates dopaminergic neurons involved in cognition, psychosis, and addiction (Bymaster et al., 2003; Dencker et al., 2011; Foster et al., 2016; Tzavara et al., 2004). Importantly, these findings have been supported by studies utilizing novel PAMs that are highly selective for the M_4_ mAChR (Bubser et al., 2014; Chan et al., 2008; Leach et al., 2010; Suratman et al., 2011). Among these, LY2033298 (LY298) was the first reported highly selective PAM of the M_4_ mAChR and displayed antipsychotic efficacy in a preclinical animal model of schizophrenia (Chan et al., 2008). Despite LY298 being one of the best characterized M_4_ mAChR PAMs, its therapeutic potential has been limited by numerous factors, including its chemical scaffold, which has been difficult to optimize with respect to its molecular allosteric parameters (Figure 1C) and variability of response between species (Suratman et al., 2011; Wood et al., 2017b). In the search for better chemical scaffolds, the PAM, VU0467154 (VU154), was subsequently discovered. VU154 showed robust efficacy in preclinical rodent models; however, it also exhibited species selectivity that prevented its clinical translation (Bubser et al., 2014). Collectively, LY298 and VU154 are exemplar tool molecules that highlight the promises and the challenges in understanding and optimizing allosteric GPCR drug activity for translational and clinical applications.

Herein, by examining the pharmacology of the PAMs LY298 and VU154 with the agonists ACh and Ipx across radioligand binding assays and two different signaling assays and analyzing these results with modern analytical methods, we determined the key parameters that describe signaling and allostery for these ligands. To investigate a structural basis for these pharmacological parameters, we used cryo-EM to determine high-resolution structures of the M_4_ mAChR in complex with a cognate G_i1_ heterotrimer and ACh and Ipx. We also determined structures of receptor complexes with Ipx co-bound with the PAMs LY298 or VU154. Moreover, because protein allostery is a dynamic process (Changeux and Christopoulos, 2016), we performed all-atom simulations using the Gaussian accelerated molecular dynamics (GaMD) enhanced sampling method (Draper-Joyce et al., 2021; Miao et al., 2015; Wang et al., 2021a) on the M_4_ mAChR using the cryo-EM structures. The structures and GaMD simulations, in combination with detailed molecular pharmacology and receptor mutagenesis experiments, provide fundamental insights into the molecular mechanisms underpinning the hallmarks of GPCR allostery. To further validate these findings, we investigated the differences in the selectivity of VU154 between the human and mouse receptors and established a structural basis for species selectivity. Collectively, these results will enable future GPCR drug discovery research and potentially lead to the development of next generation M_4_ mAChR PAMs.

## Results

### Pharmacological characterization of M_4_ mAChR PAMs with ACh and Ipx

The pharmacology of LY298 or VU154 interacting with ACh has been well characterized in binding and functional assays at the M_4_ mAChR (Bubser et al., 2014; Chan et al., 2008; Gould et al., 2016; Leach et al., 2010; Suratman et al., 2011; Thal et al., 2016). However, their pharmacology with Ipx has not been reported. Therefore, we characterized both PAMs with ACh and Ipx in binding and in two different functional assays to provide a thorough foundational comparative characterization of the pharmacological parameters of these ligands from the same study.

We first used radioligand binding assays (Figure 1—figure supplement 1A) to determine the *binding affinities* (i.e., equilibrium dissociation constants) of ACh and Ipx (K_A_) for the orthosteric site and of LY298 and VU154 (K_B_) for the allosteric site of the unoccupied human M_4_ mAChR (Figure 1D), along with the degree of *binding cooperativity* (α) between the agonists and PAMs when the two are co-bound (Figure 1E). Analysis of these experiments revealed that LY298 and VU154 have very similar binding affinities for the allosteric site with values (expressed as negative logarithms; pK_B_) of 5.65 ± 0.07 and 5.83 ± 0.12, respectively (Table 1), in accordance with previous studies (Bubser et al., 2014; Leach et al., 2011). Both PAMs potentiated the binding affinity of ACh and Ipx (Figure 1E), with the effect being greatest between LY298 and ACh (~400-fold increase in binding affinity). Comparatively, the positive cooperativity between VU154 and ACh was only 40-fold. When Ipx was used as the agonist, the binding affinity modulation mediated by both PAMs was more modest, characterized by an approximately 72-fold potentiation for the combination of Ipx and LY298, and 10-fold potentiation for the combination of Ipx and VU154. These results indicate *probe-dependent* effects (Valant et al., 2012) with respect to the ability of either PAM to modulate the affinity of each agonist (Figure 1F and G). A probe-dependent effect was also observed with the radioligand, [^3^H]-NMS, evidenced by a reduction in specific radioligand binding due to negative cooperativity between the antagonist probe and LY298, which has been previously reported (Chan et al., 2008; Leach et al., 2010; Suratman et al., 2011; Thal et al., 2016). It is important to note that binding affinity modulation is thermodynamically reciprocal at equilibrium, and the affinities of LY298 and VU154 were thus also increased in the agonist bound state (Figure 1—figure supplement 1A). This results in LY298 having a fivefold higher binding affinity than VU154 when agonists are bound (Table 1).

**Table 1.** Pharmacological parameters from radioligand binding and functional experiments.

[^3^H]-NMS saturation binding on stable M_4_ mAChR CHO cells							
Constructs		Sites per cell*			pK_D_^†^		
Human WT M_4_ mAChR		598,111 ± 43,067 (7)			9.76 ± 0.05 (7)		
Mouse WT M_4_ mAChR		21,027 ± 2188 (3)			9.76 ± 0.05 (3)		
Human D432E M_4_ mAChR		126,377 ± 10,066 (3)			9.60 ± 0.07 (3)		
Human T433R M_4_ mAChR		157,442 ± 36,658 (6)			9.64 ± 0.09 (6)		
Human V91L, D432E, T433R M_4_ mAChR		205,771 ± 20,975 (4)			9.58 ± 0.08 (4)		
**[**^**3**^**H]-NMS interaction binding assays between ACh or Ipx and LY298 or VU154 on stable M**_**4**_ **mAChR constructs in Flp-In CHO cells**							
Constructs	PAM	pK_i_ ACh ^‡^	pK_i_ Ipx ^‡^	pK_B_ PAM ^‡^	log α_ACh_ ^§^	log α_Ipx_ ^§^	
Human WT M_4_ mAChR	LY298	4.50 ± 0.06 (4)	8.30 ± 0.06 (4)	5.65 ± 0.07 (8) ^¶^	2.59 ± 0.10 (4)	1.86 ± 0.10 (4)	
	VU154	4.40 ± 0.09 (4)	8.19 ± 0.06 (8)	5.83 ± 0.11 (12) ^¶^	1.61 ± 0.13 (4)	1.03 ± 0.10 (8)	
Mouse WT M_4_ mAChR	LY298	4.52 ± 0.07 (4)	8.55 ± 0.06 (4)	5.74 ± 0.07 (8) ^¶^	1.78 ± 0.10 (4)	1.30 ± 0.11 (4)*	
	VU154	4.59 ± 0.06 (4)	8.57 ± 0.06 (3)	6.07 ± 0.09 (7) ^¶^	2.43 ± 0.10 (4)	1.75 ± 0.12 (3)*	
Human D432E M_4_ mAChR	LY298	N.T.	8.28 ± 0.04 (5)	5.86 ± 0.07 (5)	N.T.	1.59 ± 0.06 (5)	
	VU154	N.T.	8.27 ± 0.06 (6)	6.21 ± 0.12 (6)	N.T.	1.04 ± 0.09 (6)	
Human T433R M_4_ mAChR	LY298	N.T.	8.05 ± 0.08 (5)	5.04 ± 0.04 (5)*	N.T.	1.91 ± 0.11 (5)	
	VU154	N.T.	7.88 ± 0.04 (5)	5.50 ± 0.08 (5)	N.T.	1.67 ± 0.07 (5)*	
Human V91L, D432E, T433R M_4_ mAChR	LY298	N.T.	7.95 ± 0.10 (4)	5.29 ± 0.26 (4)	N.T.	1.80 ± 0.22 (4)	
	VU154	N.T.	7.89 ± 0.12 (4)	6.34 ± 0.16 (4)*	N.T.	1.35 ± 0.16 (4)	
**Gα**_**i1**_ **activation (TruPath) interaction assays between ACh or Ipx and LY298 or VU154 on transiently expressed M**_**4**_ **mAChR constructs in HEK293A cells**							
Constructs	PAM	log τ ACh**	log τ Ipx**	pK_B_ PAM ^‡^	log τ PAM**	log αβ_ACh_^††^	log αβ_Ipx_^††^
Human WT M_4_ mAChR	LY298	2.71 ± 0.14 (4)	1.49 ± 0.12 (4)	= 5.65	1.02 ± 0.03 (8) ^¶^	2.01 ± 0.14 (4)	1.96 ± 0.16 (4)
	VU154			= 5.83	–0.55 ± 0.08 (8) ^¶^	1.22 ± 0.13 (4)	0.20 ± 0.13 (4)
**pERK1/2 interaction assays between ACh or Ipx and LY298 or VU154 on stable M**_**4**_ **mAChR constructs in Flp-In CHO cells**							
Constructs	PAM	log τ ACh**	log τ Ipx**	pK_B_ PAM ^‡^	log τ_C_ PAM ^‡ ‡^	log αβ_ACh_^††^	log αβ_Ipx_^††^
Human WT M_4_ mAChR	LY298	3.27 ± 0.06 (8) ^¶^	1.74 ± 0.03 (16) ^¶^	= 5.65	1.19 ± 0.05 (12)**	2.29 ± 0.22 (4)	1.08 ± 0.28 (8)
	VU154			= 5.83	0.11 ± 0.05 (12)**	0.88 ± 0.23 (4)	0.66 ± 0.15 (8)
Mouse WT M_4_ mAChR	LY298	N.T.	N.D.	= 5.74	1.32 ± 0.07 (5)	N.T.	1.24 ± 0.12 (4)
	VU154	N.T.	N.D.	= 6.07	1.47 ± 0.08 (5) ^§ §^	N.T.	2.08 ± 0.15 (5) ^§ §^
Human D432E M_4_ mAChR	LY298	N.T.	N.D.	= 5.86	1.34 ± 0.08 (5)	N.T.	1.37 ± 0.28 (5)
	VU154	N.T.	N.D.	= 6.21	0.78 ± 0.08 (5) ^§ §^	N.T.	1.02 ± 0.15 (5)
Human T433R M_4_ mAChR	LY298	N.T.	N.D.	= 5.04	1.73 ± 0.13 (5) ^§ §^	N.T.	1.85 ± 0.28 (5)
	VU154	N.T.	N.D.	= 5.50	0.95 ± 0.12 (5) ^§ §^	N.T.	1.18 ± 0.14 (5)
Human V91L, D432E, T433R M_4_ mAChR	LY298	N.T.	N.D.	= 5.29	1.62 ± 0.09 (5) ^§ §^	N.T.	1.64 ± 0.30 (5)
	VU154	N.T.	N.D.	= 6.34	0.68 ± 0.06 (5) ^§ §^	N.T.	1.34 ± 0.11 (5) ^§ §^

Values represent the mean ± SEM with the number of independent experiments shown in parenthesis.

N.T.: not tested; N.D.: not determined; Ach, acetylcholine; Ipx: iperoxo; PAM: positive allosteric modulator.

* Number of [^3^H]-NMS binding sites per cell.

† Negative logarithm of the radioligand equilibrium dissociation constant.

‡ Negative logarithm of the orthosteric (pK_i_) or allosteric (pK_B_) equilibrium dissociation constant.

§ Logarithm of the binding cooperativity factor between the agonist (ACh or Ipx) and the PAM (LY298 or VU154).

¶ Parameter was determined in a shared global analysis between agonists.

** Logarithm of the operational efficacy parameter determined using the Operational Model of Agonism.

†† Logarithm of the functional cooperativity factor between the agonist (ACh or Ipx) and the PAM (LY298 or VU154).

‡ ‡ logτ_C_ = logarithm of the operational efficacy parameter corrected for receptor expression (methods in Appendix 1).

§ § Values from pK_B_ PAM, log α_Ipx_, log τ_C_ PAM, and log αβ_Ipx_ that are significantly different from human WT M_4_ mAChR (p<0.05) calculated by a one-way ANOVA with a Dunnett’s post-hoc test.

We subsequently used the BRET-based TruPath assay (Olsen et al., 2020) as a proximal measure of G protein activation with Gα_i1_ (Figure 1—figure supplement 1B). We also used a more amplified downstream signaling assay, extracellular signal-regulated kinases 1/2 phosphorylation (pERK1/2), that is also dependent on G_i_ activation (Figure 1—figure supplement 2A), to measure the cell-based activity of each PAM with each agonist. These signaling assays allowed us to determine the *efficacy* of the agonists (τ_A_) and the PAMs (τ_B_) (Figure 1H, Figure 1—figure supplement 2B). Importantly, efficacy (τ), as defined from the Black–Leff operational model of agonism (Black and Leff, 1983), is determined by the ability of an agonist to promote an active receptor conformation, the receptor density (B_max_), and the subsequent ability of a cellular system to generate a response (Figure 1B). Notably, in both signaling assays, the rank order of efficacy was ACh > Ipx > LY298 > VU154. We subsequently calculated the *transducer coupling coefficient* (τ/K) (Figure 1I, Figure 1—figure supplement 2C), a parameter often used as a starting point to quantify biased agonism (Kenakin et al., 2012) and that is specific to the intact cellular environment in which a given response occurs. Thus the dissociation constant (K) in the transduction coefficient subsumes the affinity for the ground state (non-bound) receptor, in addition to any isomerization states of the receptor that ultimately yield cellular responses (Kenakin and Christopoulos, 2013). Consequently, in both assays, the rank order of transducer coupling was Ipx >> ACh ~ LY298 > VU154 due to Ipx having a higher binding affinity for the receptor. Overall, these results indicate that although ACh is a more efficacious agonist than Ipx, it has lower transducer coupling coefficient. In contrast, LY298 has both better efficacy and transducer coupling coefficient than VU154 (Table 1).

The signaling assays and use of an operational model of allosterism also allowed for the determination of the *functional cooperativity* (αβ) exerted by the PAMs (Figure 1J, Figure 1—figure supplement 2D), which is a composite parameter accounting for both binding (α) and efficacy (β) modulation. Notably, VU154 displayed lower positive functional cooperativity with ACh than LY298. Strikingly, VU154 had negligible functional modulation with Ipx, in contrast to the cooperativity observed with ACh in the TruPath assay. The tenfold difference in αβ values for VU154 between ACh and Ipx highlights the dependence of the orthosteric probe used in the assay (i.e. probe dependence); on this basis, VU154 would be classified as a *‘neutral’ allosteric ligand* (not a PAM) with Ipx in the TruPath assay, that is, VU154 still binds to the allosteric site, but displays neutral cooperativity (α*β* = 1) with Ipx (Table 1).

The degree of *efficacy modulation* (β) that the PAMs have on the agonists can be calculated by subtracting the binding modulation (α) from the functional modulation (αβ) (Figure 1K, Figure 1—figure supplement 2E). A caveat of this analysis is that errors for β are higher due to the error being propagated between calculations. Ideally, the degree of efficacy modulation would be determined in an experimental system where the maximal efficacy of system is not reached by the agonists alone (Berizzi et al., 2016). Nevertheless, our analysis shows the PAMs LY298 and VU154 appear to have a slight negative to neutral effect on agonist efficacy in the G_i1_ TruPath and pERK1/2 assays (Table 1), suggesting that the predominant allosteric effect exerted by these PAMs is mediated through modulation of binding affinity.

Collectively, our extensive analysis on the pharmacology of LY298 and VU154 with ACh and Ipx offers detailed insight into the key differences between these ligands across a range of pharmacological properties: ligand binding, probe dependence, efficacy, agonist–receptor–transducer interactions, and allosteric modulation (Figure 1, Table 1). We hypothesized that structures of the human M_4_ mAChR in complex with different agonists and PAMs combined with molecular dynamic simulations could provide high-resolution molecular insights into the different pharmacological profiles of these ligands.

### Determination of M_4_R-G_i1_ complex structures

Similar to the approach used in prior determination of active-state structures of the M_1_ and M_2_ mAChRs (Maeda et al., 2019), we used a human M_4_ mAChR construct that lacked residues 242–387 of the third intracellular loop to improve receptor expression and purification, and made complexes of the receptor with G_i1_ protein and either the endogenous agonist, ACh, or Ipx. Due to the higher affinity of Ipx compared to ACh (Schrage et al., 2013), we utilized Ipx to form additional M_4_R-G_i1_ complexes with or without the co-addition of either LY298 or VU154. In all instances, complex formation was initiated by combining purified M_4_ mAChR immobilized on anti-FLAG resin with detergent solubilized G_i1_ membranes, a single-chain variable fragment (scFv16) that binds Gα_i_ and Gβ, and the addition of apyrase to remove guanosine 5′-diphosphate (Maeda et al., 2018). For this study, we used a G_i1_ heterotrimer composed of a dominant negative form of human Gα_i1_, and human Gβ_1_ and Gγ_2._ (Liang et al., 2018b). Vitrified samples of each complex were imaged using conventional cryo-TEM on a Titan Krios microscope (Danev et al., 2021).

**Figure 2.**
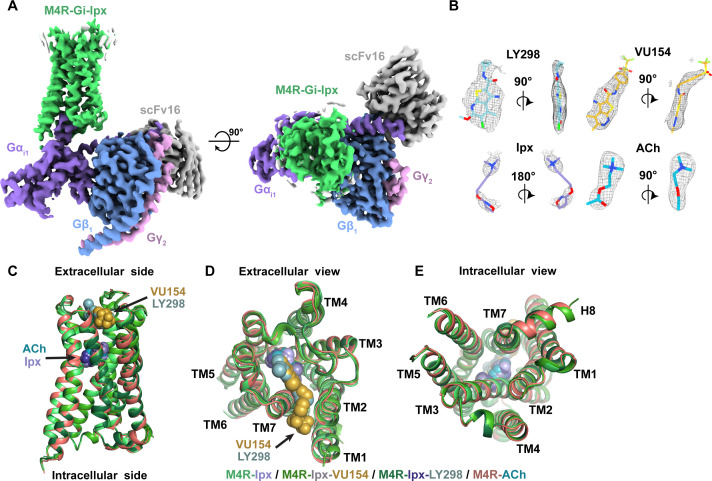
Cryo-electron microscopy (cryo-EM) structures of the M_4_R-G_i1_-scFv16 complexes. (**A**) Cryo-EM maps of Ipx-bound M_4_R-G_i1_-scFv16 complex with views from the membrane and the extracellular surface. Cryo-EM maps of the other ligand-bound structures are shown in Figure 2—figure supplement 1. (**B**) Representative EM density around the ligands in this study. EM-maps of Ipx-, LY298-Ipx-, and VU154-Ipx were set to a contour level of 0.011 and the receptor-focused map of ACh- was set to 0.32. (**C–E**) Comparison of the receptor models with bound ligands and views from the (**C**) membrane, (**D**) extracellular surface, and (**E**) intracellular surface.

The structures of ACh-, Ipx-, LY298-Ipx-, and VU154-Ipx-bound M_4_R-G_i1_ complexes were determined to resolutions of 2.8, 2.8, 2.4, and 2.5 Å, respectively (Figure 2A, Figure 2—figure supplement 1, Table 2). For the ACh-bound M_4_R-G_i1_ complex, an additional focus refinement yielded an improved map of the receptor and binding site (2.75 Å) for modeling (Figure 2—figure supplements 2 and 3). The cryo-EM density maps for all complexes were sufficient for confident placement of backbone and sidechains for most of the receptor, G_i1_, and scFv16, and the bound ligands with exception of the alkyne bond of Ipx, which was consistent with prior cryo-EM studies (Maeda et al., 2019; Figure 2B, Figure 2—figure supplement 3).

**Figure 2—figure supplement 1.**
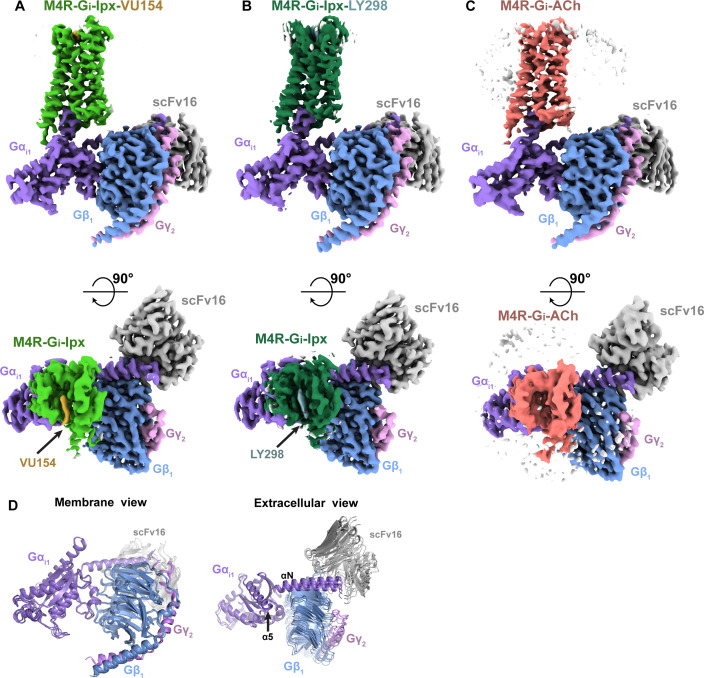
Cryo-electron microscopy (cryo-EM) structures of the M_4_R-G_i1_-scFv16 complexes. (**A–C**) Cryo-EM maps of (**A**) VU154-Ipx, (**B**) LY298-Ipx-, and (C) ACh-bound M_4_R-G_i1_-scFv16 complex with views from the membrane and the extracellular surface. The comparison of receptor models is shown in Figure 2. (**D**) Comparison of the positions of Gα_i1_Gβ_1_Gγ_2_-scFv16 from all four cryo-EM structures with views from the membrane and extracellular surface.

**Figure 2—figure supplement 2.**
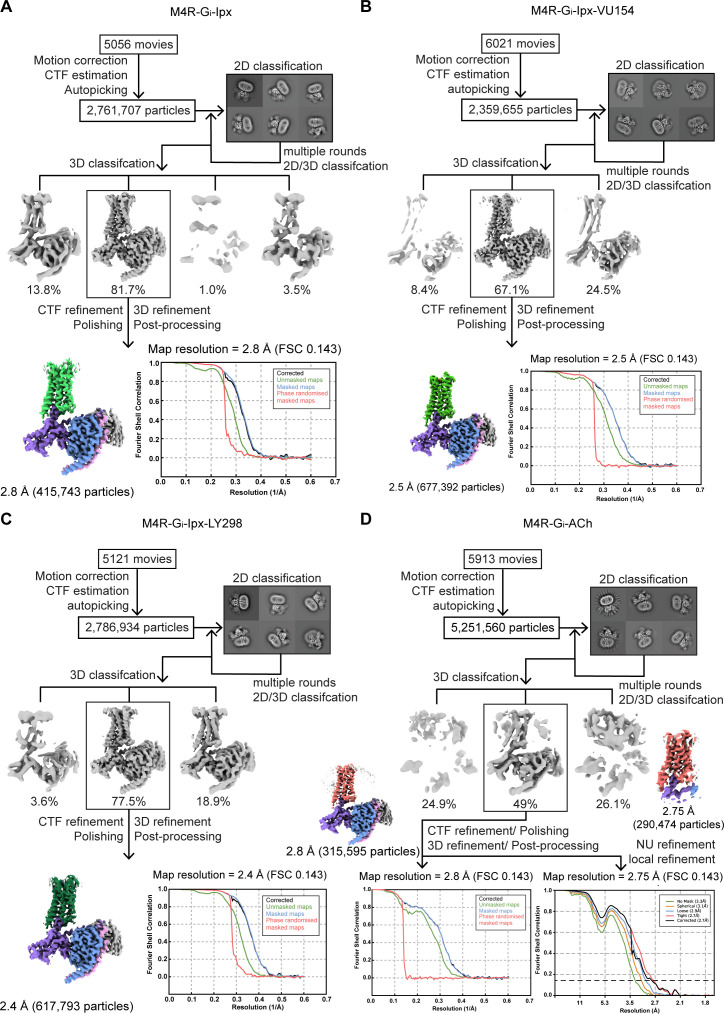
Cryo-electron microscopy (cryo-EM) data processing and analysis. (**A–D**) Flowchart of cryo-EM data processing of the (**A**) Ipx-, (**B**) VU154-Ipx-, (**C**) LY298-Ipx-, and (**D**) ACh-bound M_4_ muscarinic acetylcholine receptor (mAChR) complexes with G_i1_-scFv16 including particle selections, 2D and 3D classifications, EM density map, and the Fourier shell correlation (FSC) curves.

**Figure 2—figure supplement 3.**
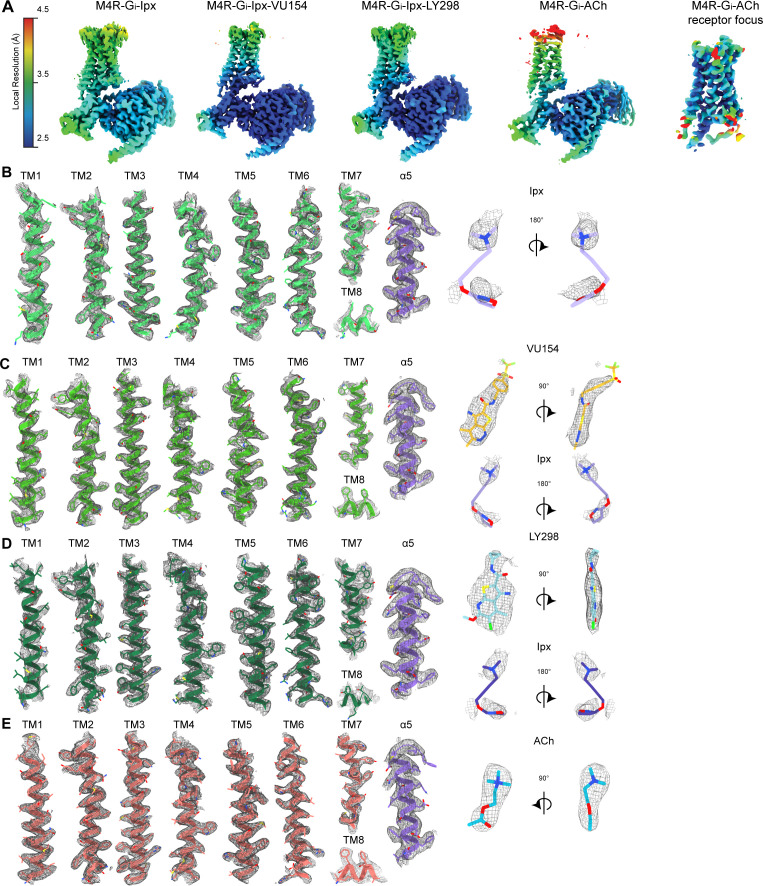
Cryo-electron microscopy (cryo-EM) density maps. (**A**) EM maps colored by local resolution. (**B–E**) Representative EM density and modeling for the 7 transmembrane (TM) helices, the C-terminus of Gα_i1_, and ligands for the (**B**) Ipx-, (**C**) VU154-Ipx-, (**D**) LY298-Ipx-, and (**E**) ACh-bound M_4_ muscarinic acetylcholine receptor (mAChR) complexes. EM-maps of Ipx-, LY298-Ipx-, and VU154-Ipx were set to a contour level of 0.011 and the receptor-focused map of ACh- was set to 0.32.

**Figure 2—figure supplement 4.**
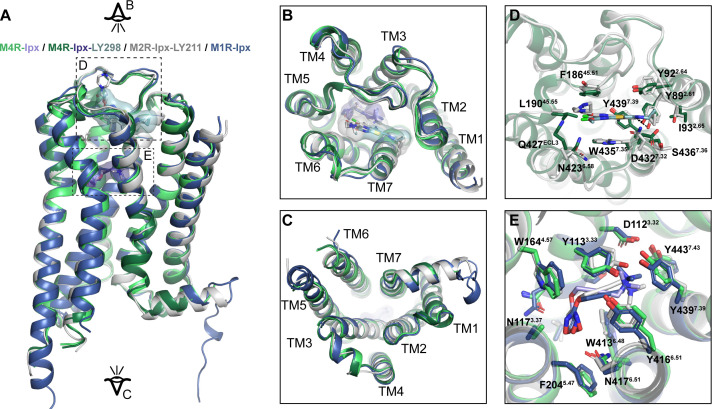
Comparison of active state muscarinic acetylcholine receptor (mAChR) structures. (**A**) Comparison of the Ipx- and LY298-Ipx-bound M_4_ mAChR structures to the prior structures of Ipx-bound M_1_ mAChR and LY2119620-Ipx-bound M_2_ mAChR cryo-EM structures. Protein Data Bank (PDB) accession codes for the M_1_ mAChR (PDB: 6OIJ) and the M_2_ mAChR (PDB: 6OIK). (**B, C**) Views from the (**B**) extracellular and (**C**) intracellular surfaces. (**D**) Comparison of the binding pose of LY2119620 at the M_2_ mAChR and LY2033298 at the M_4_ mAChR. (**E**) Comparison of the Ipx binding site residues.

**Figure 2—figure supplement 5.**
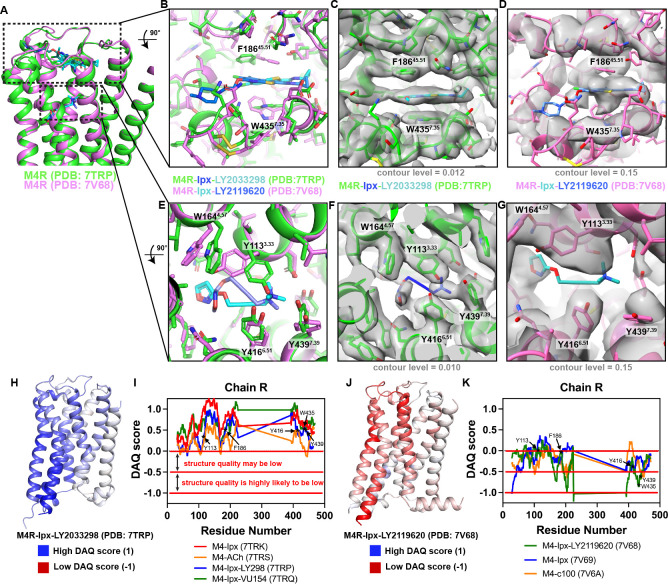
Comparison of active state M_4_ muscarinic acetylcholine receptor (mAChR) structures. (**A**) Comparison of LY298-Ipx bound M_4_ mAChR structure (PDB: 7TRP, receptor colored green, Ipx blue, and LY298 cyan) to the LY2119620-Ipx bound M_4_ mAChR structure (PDB: 7V68, receptor colored pink, Ipx cyan, and LY2119620 blue) (Wang et al., 2022). (**B–D**) View of the allosteric binding site from the top of the receptor. (**B**) Comparison of key allosteric residues F186^45.51^ and W435^7.35^ showing different positions of the residues between M_4_ mAChR structures. (**C**) Overlay of the EM map (EMD-26100, colored gray) onto the LY298-Ipx bound M_4_ mAChR structure contoured at 0.012. (**D**) Overlay of the EM map (EMD-31738, colored gray) onto the LY2119620-Ipx bound M_4_ mAChR structure contoured at 0.15. There is a lack of EM density surrounding the allosteric residues F186^45.51^ and W435^7.35^ at this level of contour and all others. (**E–G**) View of the orthosteric binding site from the top of the receptor. (**E**) Comparison of key orthosteric binding site residues. (**F**) Related to (**C**) with view from orthosteric site and the EM-map contoured at 0.010. (**G**) Related to (**D**) with view from the orthosteric site with mismodeled residues. (**H–K**) DAQ scores provide an estimation of the local quality of protein models from cryo-electron microscopy (cryo-EM) maps on a per residue basis. DAQ scores were determined from the DAQ web server using the recommended default settings (Terashi et al., 2022). (**H, J**) DAQ scores from the analysis of (**H**) the LY298-Ipx-M_4_R-G_i1_ complex and (**J**) the LY2119620-Ipx-M_4_R-G_i1_ complex mapped onto the cartoon of the receptor chain and color coded by score. A DAQ score that is positive (colored blue at values of 1) indicates a correct assignment. A DAQ score near 0 (colored white) indicates a position in the map that lacks a distinct density pattern for the assigned amino acid. DAQ scores less than 0 (colored red at –1) indicate a position that could be misassigned or poorly fit. (**I**) DAQ scores for all four M_4_ mAChR structures reported in this article with DAQ scores of each Cα atom plotted for each residue. Key orthosteric and allosteric residues are denoted by asterisks. Nearly every residue has a value above 0. (**K**) Similar to (**I**), but for all three M_4_ mAChR structures reported in Wang et al., 2022. Very few residues have a score above 0, indicating potential issues with the model and maps.

**Table 2.** Cryo-electron microscopy (cryo-EM) data collection, refinement, and validation statistics.

	M4R-G_i1_-Ipx	M4R-G_i1_-Ipx-LY298	M4R-G_i1_-Ipx-VU154	M4R-G_i1_-ACh
**Data collection & refinement**				
EMD code	26,099	26,100	26,101	26,102
Micrographs	5056	5121	6021	5913
Electron dose (e^-^/A^2^)	66	66	59.5	53.6
Voltage (kV)	300	300	300	300
Pixel size (Å)	0.83	0.83	0.83	0.83
Spot size				
Exposure time	4	4	3	5
Movie frames	76	76	75	71
K3 CDS mode	No	No	No	Yes
Defocus range (µm)	0.5–1.5	0.5–1.5	0.5–1.5	0.5–1.5
Symmetry imposed	C1	C1	C1	C1
Particles (final map)	415,743	617,793	677,392	315,595
Resolution @0.143 FSC (Å)	2.8	2.4	2.5	2.8
Refinement				
CC_map–model_	0.87	0.87	0.88	0.82
Map sharpening B factor (Å^2^)	–80.9	–60.8	–46.6	–85.1
**Model quality**				
PDB code	7TRK	7TRP	7TRQ	7TRS
R.M.S. deviations				
Bond length (Å)	0.004	0.004	0.005	0.006
Bond angles (^o^)	0.849	0.811	0.826	0.773
Ramachandran				
Favored (%)	98.38	99.14	98.02	98.10
Outliers (%)	0	0	0	0
Rotamer outliers (%)	0.11	0.21	0	0
C-beta deviations (%)	0	0	0	0
Clashscore	2.69	2.62	2.26	4.08
MolProbity score	1.06	1.05	1.00	1.19

mAChR: muscarinic acetylcholine receptor; ACh: acetylcholine; Ipx: iperoxo; FSC: Fourier shell correlation.

In all four structures, EM density beyond the top of transmembrane helix 1 (TM1) and the third intracellular loop (ICL3) of the receptor was poorly observed and not modeled. Similarly, the EM density of the α-helical domain of Gα_i1_ was poor and not modeled. These regions are highly dynamic and typically not modeled in many class A GPCR-G protein complex structures. Apart from these regions, most amino acid side chains were well resolved in the final EM density maps (Figure 2—figure supplement 3).

### Structure and dynamics of agonist binding

Recently, cryo-EM structures of M_4_R-G_i1_ complexes bound to Ipx, Ipx, and the PAM, LY2119620, and a putative novel allosteric agonist, c110, were determined (Wang et al., 2022). Surprisingly, comparison of the M_4_R-G_i1_ complex structures revealed larger differences in the position of key orthosteric and allosteric site residues than the M_1_R-G_11_ and M_2_R-G_oA_ complex structures (Figure 2—figure supplement 4). Unfortunately, the quality of density in the EM maps around the orthosteric and allosteric sites of these M_4_R-G_i1_ structures (Wang et al., 2022) was poor, resulting in several key residues being mismodeled in each site (Figure 2—figure supplement 5). Therefore, differences between the M_4_R-G_i1_ structures described herein and those by Wang et al., 2022 are highly likely to not be due to genuine differences and, as such, we compared the prior M_1_R-G_11_ and M_2_R-G_oA_ complex structures (Maeda et al., 2019) in this study.

**Figure 3.**
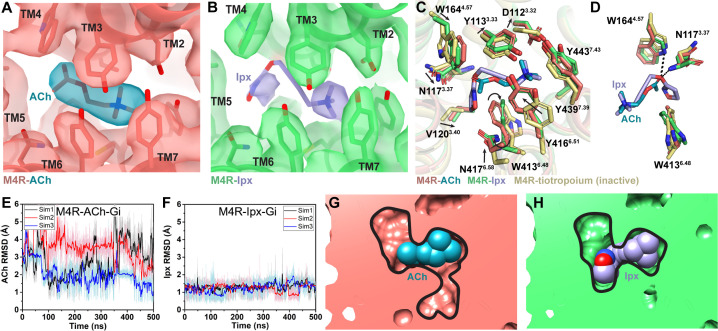
Interactions of acetylcholine (ACh) and iperoxo (Ipx) with the receptor. (**A, B**) Cryo-electron microscopy (cryo-EM) density of the (**A**) ACh- and (**B**) Ipx-bound structures. (**C, D**) Interactions at the orthosteric binding site comparing the active state ACh- and Ipx-bound structures with the inactive state tiotropium-bound structure (PDB: 5DSG). Arrows denote relative movement of residues between the inactive and active states. (**D**) Detailed interactions of ACh and Ipx. Hydrogen bonds are shown as black dashed lines. (**E, F**) Time courses from Gaussian accelerated molecular dynamics (GaMD) simulations of the ACh- and Ipx- bound M_4_R-G_i1_ cryo-EM structures, each performed with three separate replicates. Individual replicate simulations are illustrated with different colors. The heading of each plot refers to the specific model used in the simulations. Root mean square deviations (RMSDs) of (**E**) ACh and (**F**) Ipx from simulations of the cryo-EM structures. (**G, H**) Cross-sections through the ACh- and Ipx-bound structures denoting the relative size of the binding pockets outlined in black.

Overall, our M_4_R-G_i1_ complex structures are similar in architecture to that of other activated class A GPCRs, including the M_1_R-G_11_ and M_2_R-G_oA_ complexes (Figure 2—figure supplement 4). Superposition of the M_4_R-G_i1_ complexes revealed nearly identical structures with root mean square deviations (RMSD) of 0.4–0.5 Å for the full complexes and 0.3–0.4 Å for the receptors alone (Figure 2C). The largest differences occur around the extracellular surface of the receptors (Figure 2D) along with slight displacements in the position of the αN helix of Gα_i1_ and Gβ_1_, Gγ_2_, and scFv16 with respect to the receptor (Figure 2—figure supplement 1D). The EM density of side chains surrounding the ACh and Ipx binding sites (Figure 3A and B) was well resolved providing the opportunity to understand structural determinants of orthosteric agonist binding. The orthosteric site of the M_4_ mAChR, in common with the other mAChR subtypes, is buried within the TM bundle in an aromatic cage that is composed of four tyrosine residues, two tryptophan residues, one phenylalanine residue, and seven other polar and nonpolar residues (Figure 3C). Notably, all 14 of these residues are absolutely conserved across all five mAChR subtypes, underscoring the difficulty in developing highly subtype-selective orthosteric agonists (Burger et al., 2018). Both ACh and Ipx have a positively charged trimethyl ammonium ion that makes cation-π interactions with Y113^3.33^, Y416^6.51^, Y439^7.39^, and Y443^7.43^ (Figure 3C; superscript refers to the Ballesteros and Weinstein scheme for conserved class A GPCR residues; Ballesteros and Weinstein, 1995). Likewise, both ACh and Ipx have a polar oxygen atom that can form a hydrogen bond to the indole nitrogen of W164^4.57^ with the oxygen of Ipx also being in position to interact with the backbone of N117^3.37^ (Figure 3D). Mutation of any of these contact residues reduces the affinity of ACh, validating their importance for agonist binding (Leach et al., 2011; Thal et al., 2016). The largest chemical difference between ACh and Ipx is the bulkier heterocyclic isoazoline group of Ipx that makes a π-π interaction with the conserved residue W413^6.48^ (Figure 3D). The residue W413^6.48^ is part of the CWxP motif, also known as the rotamer toggle switch, a residue that typically undergoes a change in rotamer between the inactive and active states of class A GPCRs (Shi et al., 2002).

**Figure 3—figure supplement 1.**
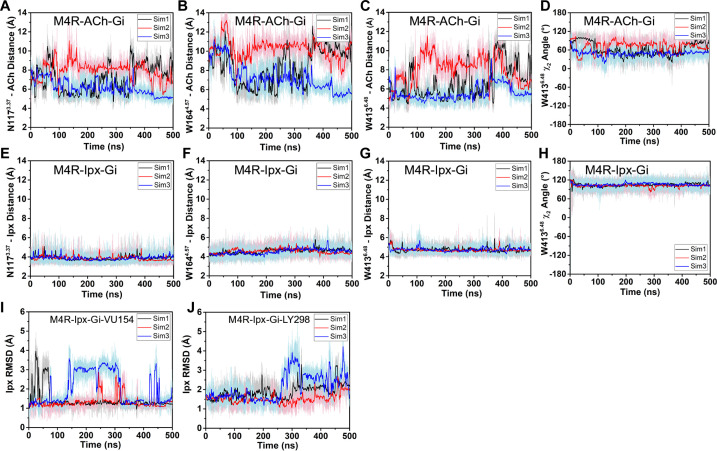
Interactions of acetylcholine (ACh) and iperoxo (Ipx) with the receptor measured during Gaussian accelerated molecular dynamics (GaMD) simulations. (**A–H**) Time courses from GaMD simulations of the ACh- and Ipx- bound M_4_R-G_i1_ cryo-electron microscopy (cryo-EM) structures, each performed with three separate replicates. Individual replicate simulations are illustrated with different colors. The heading of each plot refers to the specific model used in the simulations. The distances of interactions between ACh and Ipx with residues (**A, E**) N117^3.37^, (**B, F**) W164^4.67^, and (**C, G**) W413^6.48^, and (**D, H**) the χ_2_ angle of W413^6.48^. (**I, J**) Root mean square deviations (RMSDs) of Ipx from GaMD simulations of the PAM-Ipx-bound cryo-EM structures. See Table 3.

To investigate the structural dynamics of the M_4_ mAChR, we performed three independent 500 ns GaMD simulations on the ACh- and Ipx-bound M_4_R-G_i1_ cryo-EM structures (Table 3). GaMD simulations revealed that ACh undergoes higher fluctuations in the orthosteric site than Ipx (Figure 3E and F, Videos 1 and 2). Similarly, the interactions of N117^3.37^, W164^4.57^, and W413^6.48^ with Ipx were more stable than those with ACh (Figure 3—figure supplement 1). In the ACh-bound structure, W413^6.48^ was in a conformation matching the inactive-state tiotropium-bound structure (Figure 3C and D). GaMD simulations also showed that W413^6.48^ sampled a larger conformational space in the ACh-bound structure than in the Ipx-bound structure (Figure 3—figure supplement 1C and G). The predominate χ_2_ angle of W413^6.48^ was approximately 60^◦^ and 105^◦^ in the ACh-bound and Ipx-bound simulations, respectively, corresponding to the cryo-EM conformations.

**Table 3.** Gaussian accelerated molecular dynamics (GaMD) simulations of the M_4_ muscarinic acetylcholine receptor (mAChR).

System	Method
M4-G_i1_-Ipx (cryo-EM structure)	GaMD (3 × 500 ns)
M4-G_i1_-Ipx-VU154 (cryo-EM structure)	GaMD (3 × 500 ns)
M4-G_i1_-Ipx-LY298 (cryo-EM structure)	GaMD (3 × 500 ns)
M4-G_i1_-ACh (cryo-EM structure)	GaMD (3 × 500 ns)
M4-D432E-G_i1_-Ipx-VU154	GaMD (3 × 500 ns)
M4-T433R-G_i1_-Ipx-VU154	GaMD (3 × 500 ns)
M4-G_i1_-ACh-VU154	GaMD (3 × 500 ns)
M4-G_i1_-ACh-LY298	GaMD (3 × 500 ns)
M4-G_i1_-VU154	GaMD (3 × 500 ns)
M4-G_i1_-LY298	GaMD (3 × 500 ns)
M4-VU154	GaMD (3 × 1000 ns)
M4-LY298	GaMD (3 ×1000 ns)

**Video 1.** Movie from one Ipx-M_4_R-G_i1_ Gaussian accelerated molecular dynamics (GaMD) simulation.

**Video 2.** Movie from one ACh-M_4_R-G_i1_ Gaussian accelerated molecular dynamics (GaMD) simulation.

Located above ACh and Ipx is a tyrosine lid formed by three residues (Y113^3.33^, Y416^6.51^, and Y439^7.39^) that separate the orthosteric binding site from an extracellular vestibule (ECV) at the top of the receptor and the bulk solvent (Figure 3C). In the inactive conformation, the tyrosine lid is partially open due to Y416^6.51^ rotating away from the binding pocket to accommodate the binding of bulkier inverse agonists such as tiotropium. In contrast, mAChR agonists are typically smaller in size than antagonists and inverse agonists, and this is reflected in a contraction of the size of the orthosteric binding pocket from 115 Å^3^ when bound to tiotropium to 77 and 63 Å^3^ when bound to ACh and Ipx, respectively (Figure 3G and H; Tian et al., 2018). Together, the smaller binding pocket of Ipx and more stable binding interactions with nearby residues that include W413^6.48^ likely explain why Ipx has greater than 1000-fold higher binding affinity than ACh.

### Structure and dynamics of PAM binding and allosteric modulation of agonist affinity

**Figure 4.**
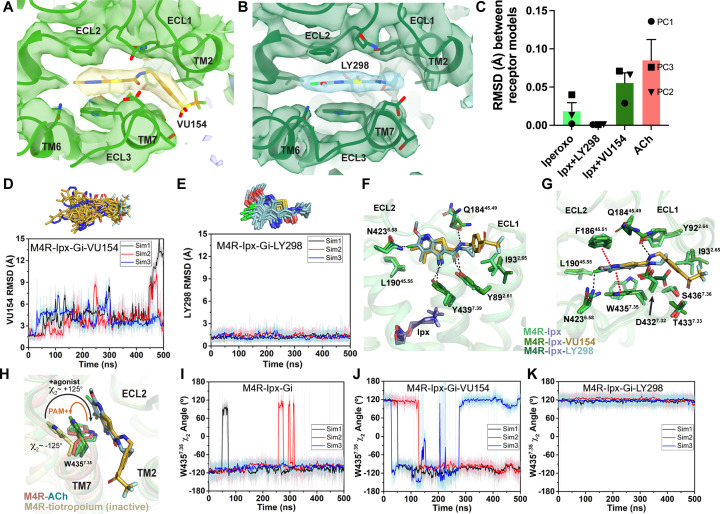
Binding and dynamics of LY298 and VU154. (**A, B**) Cryo-electron microscopy (cryo-EM) density of the (**A**) VU154- and (**B**) LY298-binding sites. (**C**) The root mean square deviations (RMSDs) between receptor models of the respective cryo-EM structures that were refined into the first and last frames of the EM maps from each principal component (PC1-PC3) of the 3D variability analysis. Values shown are mean ± SEM. (**D, E**) Top representative binding conformations of (**D**) VU154 and (**E**) LY298 obtained from structural clustering with frame populations ≥1% and time courses of the RMSDs of each positive allosteric modulator (PAM) relative to the cryo-EM structures. (**F, G**) Binding interactions of VU154 and LY298 with views from the (**F**) membrane and (**G**) extracellular surface. (**H**) Position and χ_2_ angle of W435^7.35^ in the tiotropium-, ACh-, Ipx-, VU154-Ipx-, and LY298-Ipx bound structures. (**I–K**) Time courses of the W435^7.35^ χ_2_ angle obtained from Gaussian accelerated molecular dynamics (GaMD) simulations on the (**I**) Ipx-, (**J**) VU154-Ipx-, and (**K**) LY298-Ipx-bound cryo-EM structures. See Table 3.

The M_4_R-G_i1_ structures of LY298 and VU154 co-bound with Ipx are very similar to the Ipx- and ACh-bound structures, as well as to prior structures of the M_2_ mAChR bound to Ipx and the PAM, LY2119620 (Figure 2—figure supplement 4; Kruse et al., 2013; Maeda et al., 2019). Both LY298 and VU154 bind directly above the orthosteric site in the ECV that is composed of a floor delineated by the tyrosine lid, and ‘walls’ formed by residues from TM2, TM6, TM7, ECL2, and ECL3 (Figure 4A and B). The EM density surrounding the PAM binding site and the ECV of the M_4_ mAChR were clearly resolved with one exception; in the VU154-bound structure, the EM density begins to weaken around the trifluoromethylsulfonyl moiety (Figure 2B, Figure 4B). This was likely due to the moiety’s ability to freely rotate and a lack of strong interactions with the receptor.

**Figure 4—figure supplement 1.**
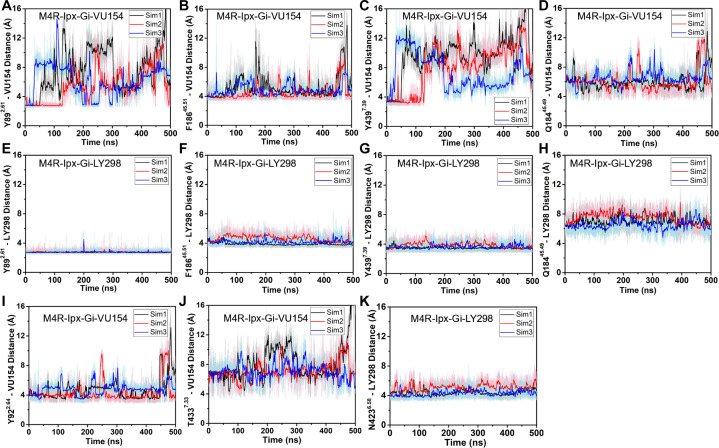
Gaussian accelerated molecular dynamics (GaMD) simulations of LY293 and VU154 binding. (**A–K**) Time courses from three 500 ns GaMD simulations using the (**A–D**) VU154-Ipx- and (**E–H**) LY298-Ipx-bound cryo-electron microscopy (cryo-EM) structures. Distances between the interactions of VU154 and LY298 with residues (**A, E**) Y89^7.39^, (**B, F**) F186^45.51^, (**C, G**) Y439^7.39^, and (**D, H**) Q184^45.49^. (**I, J**) Distance between (**I**) Y92^2.64^ and (**J**) T433^7.33^ to VU154 from GaMD simulations of the VU154-Ipx-M4R-G_i1_ structure. (**K**) Distance between N423^6.58^ and the fluorine atom of LY298 from GaMD simulations of the LY298-Ipx-M4R-G_i1_ structure. See Table 3.

**Figure 4—figure supplement 2.**
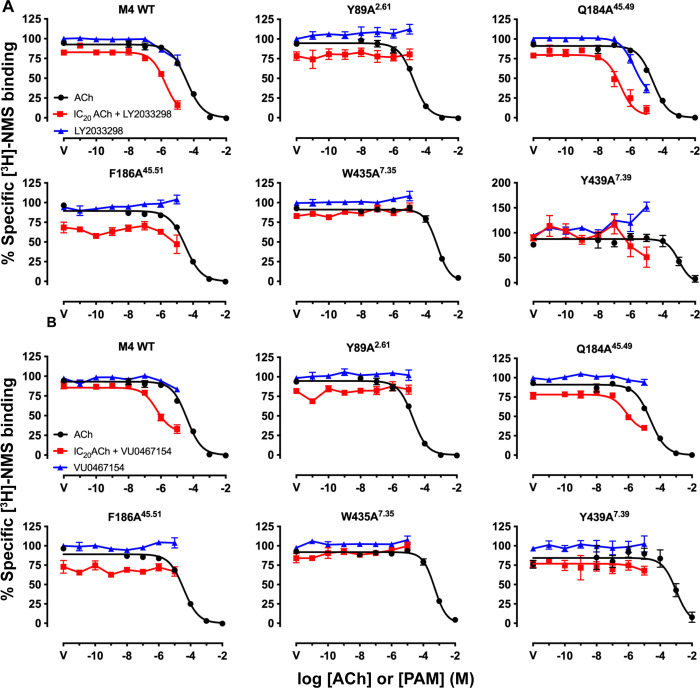
Key residues for the binding of LY298 and VU154 at the human M4 muscarinic acetylcholine receptor (mAChR). (**A, B**) Competition binding with a fixed concentration of [^3^H]-NMS and increasing concentrations of acetylcholine (ACh) (black circles), (**A**) LY298 or (**B**) VU154 (blue circles), and LY298 or VU154 in the presence of an IC_20_ concentration of ACh (red squares). Curves drawn through the points represent a global fit of an extended ternary complex model. Data points represent the mean ± SEM of three or more independent experiments performed in duplicate. Similar data were observed for competition binding with iperoxo (Ipx) instead of ACh. See Table 4. Figure 4—figure supplement 2—source data 1.Related to Figure 4—figure supplement 2.

**Figure 4—figure supplement 3.**
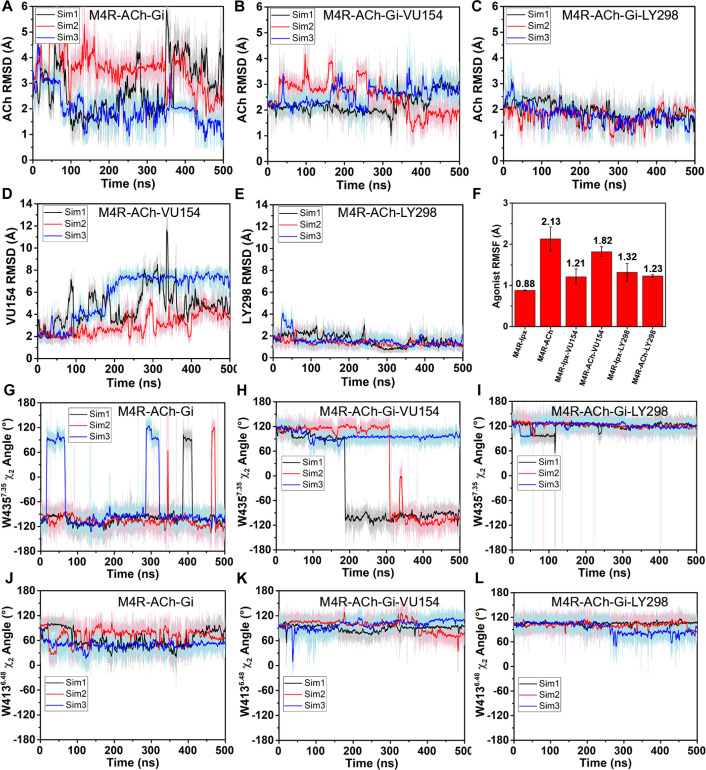
Gaussian accelerated molecular dynamics (GaMD) simulations of M_4_R complexes with acetylcholine (ACh). (**A–L**) Time courses from GaMD simulations, each performed with three separate replicates. Individual replicate simulations are illustrated with different colors. The heading of each plot refers to the specific model used in the simulations. See Table 3. (**A–C**) Root mean square deviations (RMSDs) of ACh from simulations of the (**A**) cryo-electron microscopy (cryo-EM) structure or (**B, C**) positive allosteric modulator (PAM) docked models. (**D, E**) RMSDs of VU154 and LY298 from the ACh-bound M_4_ mAChR simulations. (**F**) Bar graph of the root mean fluctuations of the agonists iperoxo (Ipx) or ACh across the GaMD simulations of the M_4_-G_i1_ complexes with or without the PAMs. Values shown are mean ± SEM, n = 3. (**G–L**) Time course of the ACh-bound M_4_-G_i1_ simulations illustrating variances in the (**G–I**) W435^7.35^ χ_2_ angle and (**J–L**) the W413^6.48^ χ_2_ angle.

Given the overall similarities revealed by our four cryo-EM structures, we examined whether there were further differences in the dynamics between the PAM-bound structures by performing a 3D multivariance analysis (3DVA) of the principal components of motion within the Ipx-, LY298-Ipx, VU154-Ipx, and ACh-bound M_4_R-G_i1_ cryo-EM data sets using Cryosparc (Punjani and Fleet, 2021); a similar analysis performed previously on cryo-EM structures of class A and class B GPCRs provided important insights into the allosteric motions of extracellular domains and receptor interactions with G proteins (Josephs et al., 2021; Liang et al., 2020; Mobbs et al., 2021; Zhang et al., 2020).

In the 3DVA of the Ipx-bound complex, the M_4_ mAChR appeared less flexible than the receptor in the ACh-bound complex (Videos 3 and 4) consistent with Ipx having a higher binding affinity and more stable pose during the GaMD simulations (Figure 3E and F). The LY298-Ipx-bound complex appeared similar to the Ipx-bound complex with LY298 being bound in the ECV (Video 5). In contrast, the 3DVA of the VU154 structure had more dynamic movements in the allosteric pocket that could reflect partial binding of VU154 (Video 6). This observation was in line with our findings that VU154 had lower binding modulation (Figure 1E) and functional modulation with agonists than LY298 (Figure 1J, Figure 1—figure supplement 2D, Table 1). To quantify the differences from the 3DVA, we rigid body fitted and refined the respective M_4_R-G_i1_ models into the first and last frames of the EM maps from each principal component of the 3DVA and then calculated the RMSD between the receptor models (Figure 4C). In agreement with our prior observations, the VU154-Ipx-bound and ACh-bound complexes had greater RMSDs with values of 0.06 and 0.09 Å, respectively. Comparatively, the Ipx-bound and LY298-Ipx-bound complexes had lower RMSD values of 0.02 and 0.001 Å, respectively. The results of the 3DVA do not represent *bona fide* measures of receptor dynamics, rather they are suggestive of differences between the collected data sets that led to the structures. To support these findings, we compared the GaMD simulations of all four cryo-EM structures (Table 3). Notably, VU154 underwent considerably higher fluctuations than LY298 with RMSDs ranging from 1.5 to 15 Å for VU154 (Video 7) and 0.8–2.1 Å for LY298 (Video 8) relative to the cryo-EM structures (Figure 4D and E). Therefore, the GaMD simulations corroborate our 3DVA results and suggest that complexes bound to agonists with high affinity or co-bound with agonists and PAMs with high positive cooperativity will exhibit lower dynamic fluctuations.

**Video 3.** 3D variability analysis of the Ipx-M_4_R-G_i1_ cryo-electron microscopy (cryo-EM) structure.

**Video 4.** 3D variability analysis of the ACh-M_4_R- G_i1_ cryo-electron microscopy (cryo-EM) structure.

**Video 5.** 3D variability analysis of the LY298-Ipx-M_4_R- G_i1_ cryo-electron microscopy (cryo-EM) structure.

**Video 6.** 3D variability analysis of the VU154-Ipx-M_4_R- G_i1_ cryo-electron microscopy (cryo-EM) structure.

**Video 7.** Movie from one VU154-Ipx-M_4_R-G_i1_ Gaussian accelerated molecular dynamics (GaMD) simulation.

**Video 8.** Movie from one LY298-Ipx-M_4_R-G_i1_ Gaussian accelerated molecular dynamics (GaMD) simulation.

To investigate why the binding of LY298 was more stable than VU154, we examined the ligand interactions with the receptor. There are three key binding interactions that are shared between both PAMs and the M_4_ mAChR: (1) a three-way π-stacking interaction between F186^45.51^ (ECL2 residues have been numbered 45.X denoting their position between TM4 and TM5 with X.50 being a conserved cysteine residue), the aromatic core of the PAMs, and W435^7.35^; (2) a hydrogen bond between Y439^7.39^ of the tyrosine lid and the primary amine of the PAMs; and (3) a hydrogen bond between Y89^2.61^ and the carbonyl oxygen of the PAMs (Figure 4F and G). While these interactions are conserved for both PAMs in the consensus cryo-EM maps, during GaMD simulations these interactions were more stable with LY298 than VU154 (Figure 4H–K, Figure 4—figure supplement 1). The importance of these interactions was validated pharmacologically (Figure 4—figure supplement 2, Table 4), whereby mutation of any of these residues completely abolished the binding affinity modulation mediated by LY298 and VU154 at the M_4_ mAChR with both Ipx and ACh as agonists.

**Table 4.** Pharmacological parameters of LY298 and VU154 at key M_4_ muscarinic acetylcholine receptor (mAChR) mutants.

[^3^H]-NMS saturation binding on stable M_4_ mAChR Flp-In CHO cells									
Constructs			Sites per cell*				pK_D_†		
Human WT M_4_ mAChR (from Table 1)			598,111 ± 43,067 (7)				9.76 ± 0.05 (7)		
Y89A^2.61^			32,674 ± 4174 (4)				9.88 ± 0.06 (4)		
Q184A^45.49^			88,728 ± 3056 (3)				9.99 ± 0.06 (3)		
F186A^45.51^			36,907 ± 4170 (4)				9.75 ± 0.16 (4)		
W435A^7.35^			34,861 ± 3510 (3)				9.81 ± 0.22 (3)		
Y439A^7.39^			42,690 ± 4547 (3)				8.31 ± 0.14 (3)		
**[^3^H]-NMS interaction binding assays between ACh or Ipx and LY298 or VU154 on stable M_4_ mAChR constructs in Flp-In CHO cells**									
Constructs	PAM	pK_i_ ACh‡		pK_i_ Ipx‡	pK_B_ PAM‡	log α_ACh_§		log α_Ipx_§	log α_NMS_¶
Human WT M_4_	LY298	5.09 ± 0.07 (7)		8.54 ± 0.04 (11)	= 5.65	1.57 ± 0.11		1.71 ± 0.09	= 0
	VU154	5.06 ± 0.05 (7)		8.54 ± 0.03 (11)	= 5.83	1.44 ± 0.07		1.11 ± 0.06	= 0
Y89A^2.61^	LY298	5.25 ± 0.05 (6)		8.48 ± 0.05 (6)	N.D.	N.D.		N.D.	N.D.
	VU154	5.27 ± 0.05 (6)		8.47 ± 0.05 (6)	N.D.	N.D.		N.D.	N.D.
Q184A^45.49^	LY298	5.24 ± 0.06 (6)		8.74 ± 0.04 (10)	6.23 ± 0.06	1.28 ± 0.13		1.27 ± 0.11	–1.10 ± 0.07
	VU154	5.28 ± 0.05 (6)		8.69 ± 0.04 (10)	5.87 ± 0.17	1.07 ± 0.09		0.81 ± 0.07	= 0
F186A^45.51^	LY298	4.91 ± 0.05 (6)		8.12 ± 0.05 (8)	N.D.	N.D.		N.D.	N.D.
	VU154	4.91 ± 0.05 (6)		8.12 ± 0.05 (8)	N.D.	N.D.		N.D.	N.D.
W435^7.35^	LY298	3.79 ± 0.07 (7)		6.88 ± 0.07 (7)	N.D.	N.D.		N.D.	N.D.
	VU154	3.79 ± 0.07 (7)		6.88 ± 0.07 (7)	N.D.	N.D.		N.D.	N.D.
Y439A^7.39^	LY298	3.23 ± 0.22 (8)		5.36 ± 0.25 (8)	N.D.	N.D.		N.D.	N.D.
	VU154	3.23 ± 0.22 (8)		5.36 ± 0.25 (8)	N.D.	N.D.		N.D.	N.D.

Values represent the mean ± SEM with the number of independent experiments shown in parenthesis.

N.D.: not determined; ACh: acetylcholine; Ipx: iperoxo; PAM: positive allosteric modulator.

* Number of [^3^H]-NMS binding sites per cell.

† Negative logarithm of the radioligand equilibrium dissociation constant.

‡ Negative logarithm of the orthosteric (pK_i_) or allosteric (pK_B_) equilibrium dissociation constant. pK_i_ values for ACh and Ipx are shared at each M4 mAChR construct. pK_B_ values for the PAMs at Q184A are shared across the agonist data sets.

§ Logarithm of the binding cooperativity factor between the agonist (ACh or Ipx) and the PAM (LY298 or VU154).

¶ Logarithm of the binding cooperativity factor between the [^3^H]-NMS and the PAM (LY298 or VU154).

A potential fourth interaction was observed with residue Q184^45.49^ and the amide nitrogen of the PAMs; however, the GaMD simulations suggest that this interaction is relatively weak (Figure 4—figure supplement 1D and H), consistent with the fact that mutation of Q184^45.49^ to alanine had no effect on the binding affinity modulation of LY298 or VU154 (Figure 4—figure supplement 2, Table 4). In addition, each PAM had at least one potential unique binding interaction with the receptor (Figure 4F and G). For LY298, this is an interaction between the fluorine atom and N423^6.58^ that appeared to be stable during simulation and, when mutated to alanine reduced the binding modulation of LY298 (Figure 4—figure supplement 1K, Figure 4—figure supplement 2, Table 4; Thal et al., 2016). For VU154, there were two additional possible hydrogen bonding interactions with residues Y92^2.64^ and T433^7.33^ (Figure 4G); however, these interactions were highly fluctuating during GaMD simulations, suggesting they were – at best – transient interactions (Figure 4—figure supplement 1I and J). Finally, W435^7.35^ is a key residue in the ECV that changes from a planar rotamer in the agonist-bound structures to a vertical rotamer that π stacks against the PAMs (Figure 4H). In GaMD simulations of the Ipx-bound structure, W435^7.35^ is predominantly in a planar conformation that corresponds to its conformation in the cryo-EM structure (Figure 4I). In contrast, the binding of LY298 stabilizes W435^7.35^ into a vertical position (Figure 4K). However, in the VU154-bound receptor, W435^7.35^ appears to alternate between the planar and vertical positions, consistent with VU154 having a less stable binding pose (Figure 4J). These results indicate that the binding of LY298 is more stable than VU154 due to LY298 being able to form stable binding interactions with key residues in the ECV. This provides a likely explanation for why LY298 was able to exert greater positive binding cooperativity on orthosteric agonists than VU154.

### A molecular mechanism of probe dependence

As highlighted above, PAMs, LY298 and VU154, displayed stronger allosteric binding affinity modulation with ACh than Ipx, an example of probe dependence (Figure 1E, Table 1). These findings are in accord with previous studies where we identified probe dependence in the actions of LY298 when tested against other orthosteric agonists (Chan et al., 2008; Suratman et al., 2011). To investigate a mechanism for probe dependence at the M_4_ mAChR, we performed GaMD simulations with LY298 and VU154 co-bound with ACh by replacing Ipx with ACh in the corresponding cryo-EM structures (Table 3, Figure 4—figure supplement 3). In the absence of PAM, ACh was more dynamic than Ipx with root-mean-square fluctuations (RMSF) of 2.13 Å versus 0.88 Å, reflective of the fact Ipx binds with higher affinity than ACh (Figure 4—figure supplement 3F). In the presence of LY298 or VU154, the dynamics of ACh binding was decreased, with RMSFs reduced to 1.23 Å and 1.82 Å, respectively, and with LY298 having the greatest effect (Figure 4—figure supplement 3F). This is in line with LY298 having more cooperativity with ACh than VU154 (Figure 1E). In comparison to ACh, there was a modest increase in the dynamics of Ipx with the addition of LY298 or VU154, likely reflecting the fact Ipx binding to the receptor was already stable (Figure 3—figure supplement 1I and J, Figure 4—figure supplement 3F). These results provide a plausible mechanism for probe dependence, at least with regard to differences in the magnitude of the allosteric effect depending on the ligand bound. Namely, PAMs manifest higher cooperativity when interacting with agonists, such as ACh, that are inherently less stable on their own when bound to the receptor, in contrast to more stable ligands such as Ipx.

### Structural and dynamic insights into orthosteric and allosteric agonism

**Figure 5.**
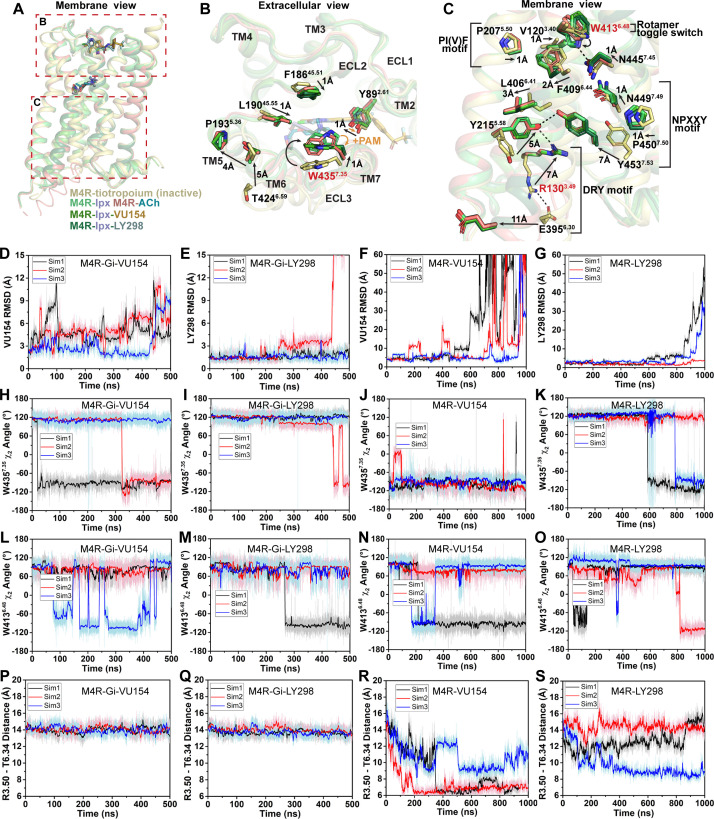
Structural and dynamic insights into orthosteric and allosteric agonism. (**A**) Cartoon of the receptor models indicating regions of interest for panels (**B, C**) shown within the red boxes. (**B**) View of the tiotropium-bound, agonist-bound, and positive allosteric modulator (PAM)-agonist-bound conformations from the extracellular surface. (**C**) Membrane view of residues and activation motifs involved in signaling. Residues colored red in (**B, C**) indicate residues of investigated in Gaussian accelerated molecular dynamics (GaMD) simulations. (**D–G**) Time course of the root mean square deviations (RMSDs) of the PAMs (**D, E**) from GaMD simulations of the M_4_R bound to G protein and no orthosteric agonist, (**F, G**) and in the absence of both G protein and agonist. (**H–K**) Similar to (**D–G**) the time courses of (**H–K**) the W435^7.35^ χ_2_ angle, (**L–O**) the W413^6.48^ χ_2_ angle, and (**P–S**) the TM3-TM6 distance measured by distance between R130^3.50^ and T399^6.34^. See Table 3.

In addition to the ability to allosterically modulate the function of orthosteric ligands, it has become increasingly appreciated that allosteric ligands may display variable degrees of direct agonism in their own right, over and above any allosteric modulatory effects (Changeux and Christopoulos, 2016). Prior studies have established that the activation process of GPCRs involves conformational changes that extend from the extracellular domains through to the intracellular surface (Nygaard et al., 2009). Comparison of the active state ACh-, Ipx-, LY298-Ipx-, and VU154-Ipx-bound M_4_R-G_i1_ structures to the inactive state tiotropium-bound M_4_ mAChR structure (Protein Data Bank accession 5DSG) (Thal et al., 2016) thus affords an opportunity to gain new insights into the activation process mediated by multiple orthosteric agonists in the presence and absence of two different PAMs that display high (LY298) and low (VU154) degrees of direct allosteric agonism (Figures 1H and 5A–C).

As discussed previously, agonist binding decreases the size of the orthosteric binding site (Figure 3G and H). The primary driver of this decrease was the tyrosine lid residue Y416^6.51^, which underwent a large rotation toward Y113^3.33^ creating a hydrogen bond that seals off the tyrosine lid (Figure 3C). The closure of the tyrosine lid was further reinforced by a change in the rotamer of W435^7.35^ to a planar position that sits parallel to the tyrosine lid allowing for a π-π interaction with Y416^6.51^ and a positioning of the indole nitrogen of W435^7.35^ to potentially form a hydrogen bond with the hydroxyl of Y89^2.61^ (Figure 5B). The contraction of the orthosteric pocket by the inward movement of Y416^6.51^ also led to a contraction of the ECV with a 5 Å inward movement of the top of TM6 and ECL3. As a consequence, the top of TM5 was displaced outward by 4 Å forming a new interface between TM5 and TM6 that was stabilized by a hydrogen bond between T424^6.59^ and the backbone nitrogen of P193^5.36^ along with aromatic interactions between F197^5.40^ and F425^6.60^ (Figure 5B). These interactions were specific to the active state structures and appear to be conserved as they were also present in the M_1_ and M_2_ mAChR active state structures (Maeda et al., 2019). In addition to the movements of TM5 and TM6, there was a smaller 1 Å inward movement of ECL2 (Figure 5B). The binding of LY298 and VU154 had a minimal impact on the conformation of most ECL residues, implying that the reorganization of residues in the ECV by orthosteric agonists contributes to the increased affinity of the PAMs (Figure 1G). There was a slight further inward shift of ECL2 toward the PAMs to facilitate the 3-way π-stacking interaction with F186^45.51^ and W435^7.35^. In addition, in the PAM-bound structures, Y89^2.61^ rotated away from its position in the ACh- and Ipx-bound structures either due to a loss of an interaction with W435^7.35^ or to form a better hydrogen bond with the carbonyl oxygen of the PAMs (Figure 5B).

Below the orthosteric binding site are several signaling motifs that are important for the activation of class A GPCRs, including the PIF motif (Rasmussen et al., 2011; Wacker et al., 2013), the Na^+^ binding site (Liu et al., 2012a; White et al., 2018), the NPxxY motif (Fritze et al., 2003), and the DRY motif (Figure 5C; Ballesteros et al., 2001). The conformations of these activation motifs were very similar across all four active-state M_4_ mAChR structures and were consistent with the position of these motifs across other active-state class A GPCR structures (Zhou et al., 2019). Collectively, all of the described activation motifs facilitate an 11 Å outward movement of TM6 that typifies GPCR activation and creation of the G protein binding site. In comparison to the ECV residues (Figure 5B), beyond the rotamer toggle switch residue W413^6.48^, there are no discernible differences between the agonist and PAM-agonist-bound structures, suggesting a shared activation mechanism for residues below W413^6.48^ (Figure 5C).

As indicated above, LY298 also displays robust allosteric agonism in comparison to VU154 (Figure 1H, Figure 1—figure supplement 2B). To probe whether the allosteric agonism of LY298 could be related to its ability to better stabilize the M_4_ mAChR in an active conformation in comparison to VU154, we performed additional GaMD simulations on the LY298-Ipx- and VU154-Ipx-bound M_4_R-G_i1_ structures with the agonist Ipx removed (3 × 500 ns) and with both Ipx and the G protein removed (3 × 1000 ns) (Figure 5D–S, Table 3). In GaMD simulations, LY298 underwent lower RMSD fluctuations than VU154 before dissociating from the receptor (Figure 5D–G). Similarly, the conformations of W435^7.35^ and W413^6.48^ were better stabilized in the LY298-Ipx-bound systems, indicating that LY298 more strongly promotes an active receptor conformation (Figure 5H–K). In the presence of the G protein, both PAMs stabilized an active conformation of the receptor based on the distances between TM3 and TM6 (Figure 5P and Q). Upon removal of the G protein, the VU154-bound M_4_ mAChR quickly transitioned toward the inactive conformation, while the LY298-bound M_4_ mAChR was more resistant to deactivation in the GaMD simulations (Figure 5R and S). This observation supports LY298 having greater efficacy than VU154 (Table 1) as it better stabilizes the active conformation of the M_4_ mAChR. Overall, the GaMD simulations show that in the absence of agonist alone, or agonist and G protein, LY298 better stabilizes activation motifs from the top of the receptor (W435^7.35^) all the way down to the intracellular G protein binding pocket (DRY-TM6), providing mechanistic insights into the function of LY298 as a stronger PAM-agonist than VU154.

### Structural insights into allosteric modulation of agonist signaling

**Figure 6.**
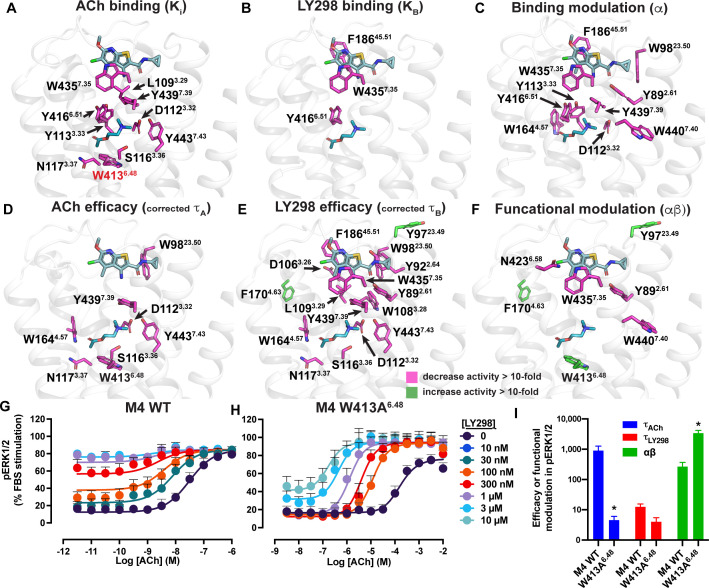
Residues involved in binding, agonism, and modulation of acetylcholine (ACh) and LY298. (**A–F**) M_4_ muscarinic acetylcholine receptor (mAChR) alanine point mutations that increase (green colored sticks) or decrease (pink colored sticks) (**A**) ACh binding, (**B**) LY298 binding, (**C**) binding modulation between ACh and LY298, (**D**) ACh efficacy, (**E**) LY298 efficacy, (**F**) and functional modulation by values more than tenfold. Efficacy values are corrected for receptor expression (Gregory et al., 2010) using receptor expression data from Thal et al., 2016. Quantitative data used to identify key residues are from both the current study and previous studies as summarized in Table 5 (Leach et al., 2011; Nawaratne et al., 2010; Thal et al., 2016). (**G–I**) pERK1/2 concentration response curves for interaction of ACh and LY298 at (**G**) WT and (**H**) W413A^6.48^ M_4_ mAChR with (**I**) values of efficacy and functional modulation. *Indicates statistical significance (p<0.05) relative to WT as determined by a one-way ANOVA with a Dunnett’s post-hoc test that includes the other M_4_ mAChR mutants. Data shown are mean ± SEM from three or more experiments performed in duplicate with the pharmacological parameters determined from a global fit of the data.

In a previous study, we characterized over 40 distinct mutations of M_4_ mAChR residues that span from the orthosteric site up to the extracellular surface (Table 5; Leach et al., 2011; Nawaratne et al., 2010; Thal et al., 2016). As expected, these studies revealed that mutation of residues around the orthosteric and allosteric sites often resulted in a reduction in the binding affinity of either ACh or LY298 at their respective binding sites, though the allosteric site was typically less affected (Figure 6A and B, Table 5). In contrast, the binding affinity modulation between ACh and LY298 was largely affected by mutation of aromatic residues that link the orthosteric and allosteric sites (Figure 6C), implying a network of residues that were responsible for transmitting binding cooperativity between these two sites (Thal et al., 2016). Analyzing unpublished data from prior studies allowed an examination of the signaling efficacy of ACh (τ_A_) and LY298 (τ_B_), but also the functional cooperativity (αβ) in the context of active state structures of the co-complexes (Figure 6D–F, Figure 6—figure supplement 1, Table 5).

**Figure 6—figure supplement 1.**
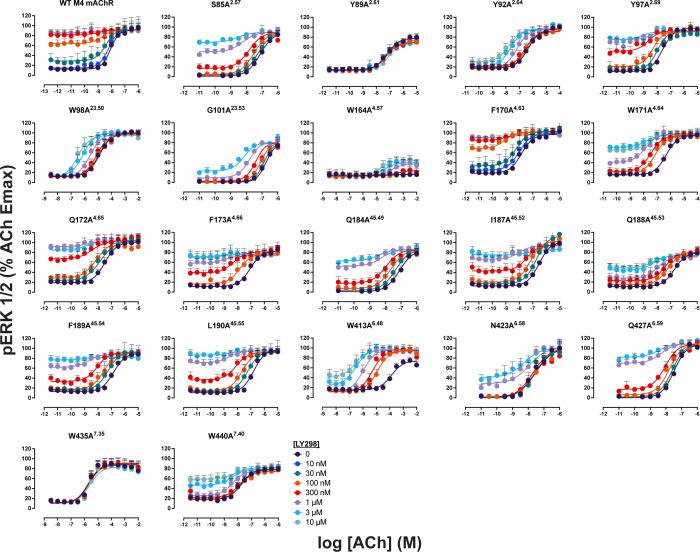
Concentration–response curves between acetylcholine (ACh) and LY298 at M_4_ muscarinic acetylcholine receptor (mAChR) mutants. Concentration–response curves of an interaction between ACh and LY298 in pERK1/2 at the WT human M_4_ mAChR and mutants characterized in this study. Parameters of curve fits are in Table 5. Data are the mean ± SEM from three or more experiments performed in duplicate with the pharmacological parameters determined from a global fit of the data.

**Figure 6—figure supplement 2.**
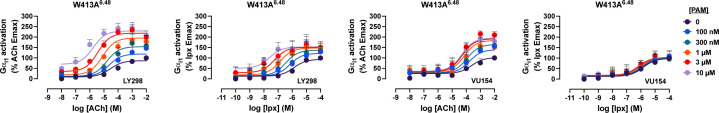
Interaction assays of agonists and positive allosteric modulators (PAMs) at the W413A^6.48^ M_4_ muscarinic acetylcholine receptor (mAChR) in a TruPath assay. Concentration–response curves of an interaction between the agonists acetylcholine (ACh) or iperoxo (Ipx) with the PAMs LY298 or VU154 at the W413A^6.48^ M_4_ mAChR in a TruPath assay. Parameters of curve fits are in Table 5. Data are the mean ± SEM from three or more experiments performed in duplicate with the pharmacological parameters determined from a global fit of the data.

**Table 5.** Pharmacological parameters of M_4_ muscarinic acetylcholine receptor (mAChR) mutants.

	pERK1/2 interaction assays*			[^3^H]-QNB interaction binding assays^†^			Study
**Constructs**	**log τ**_**C**_ **ACh** ^**‡**^	**log τ**_**C**_ **LY298** ^‡^	**log αβ** ^§^	**pK**_**i**_ **ACh** ^¶^	**pK**_**B**_ **LY298** ^¶^	**log α** **	
WT M_4_ mAChR	2.96 ± 0.14 (4)	1.10 ± 0.09	2.43 ± 0.14	4.51 ± 0.15	4.89 ± 0.12	1.97 ± 0.11	Current/Thal^††^
S85A^2.57^	3.15 ± 0.11(4)	0.91 ± 0.07	1.75 ± 0.09	4.09 ± 0.11	5.44 ± 0.14	1.43 ± 0.06	Current/Thal
Y89A^2.61^	2.53 ± 0.17 (5)	= –3	–0.43 ± 0.27*	5.07 ± 0.45	5.36 ± 0.03	–0.13 ± 0.08 ^‡ ‡^	Current/Thal
Y92A^2.64^	2.26 ± 0.15 (4)	–0.06 ± 0.16*	2.25 ± 0.11	4.15 ± 0.25	4.53 ± 0.15	1.2 ± 0.19 ^‡ ‡^	Current/Thal
I93T, I94V, K95I	2.57 ± 0.11	2.27 ± 0.19 ^‡ ‡^	N.T.	4.69 ± 0.11	4.82 ± 0.36	2.14 ± 0.17 ^‡ ‡^	Nawaratne ^§ §^
I93T^2.65^	2.34 ± 0.09	2.38 ± 0.22 ^‡ ‡^	N.T.	4.97 ± 0.04	5.36 ± 0.09	2.42 ± 0.16 ^‡ ‡^	Nawaratne
I94V^2.66^	2.34 ± 0.09	1.24 ± 0.09	N.T.	4.71 ± 0.06	5.17 ± 0.08	1.74 ± 0.07	Nawaratne
K95I^2.67^	2.00 ± 0.07	0.61 ± 0.09 ^‡ ‡^	N.T.	4.86 ± 0.05	5.20 ± 0.14	1.24 ± 0.04 ^‡ ‡^	Nawaratne
Y97A^23.49^	2.94 ± 0.11 (4)	2.19 ± 0.07*	3.43 ± 0.12*	4.69 ± 0.17	4.25 ± 0.10 ^‡ ‡^	2.33 ± 0.12	Current/Thal
W98A^23.50^	1.45 ± 0.15* (5)	= –3	2.29 ± 0.10	3.65 ± 0.11 ^‡ ‡^	4.39 ± 0.04	0.73 ± 0.07 ^‡ ‡^	Current/Thal
G101A^23.53^	2.58 ± 0.09 (4)	0.43 ± 0.08*	2.10 ± 0.07	4.37 ± 0.19	5.03 ± 0.17	1.47 ± 0.01	Current/Thal
D106A^3.26^	1.24 ± 0.11	= –3	N.T.	3.95 ± 0.09 ^‡ ‡^	5.29 ± 0.11	1.51 ± 0.15	Leach ^¶ ¶^
W108A^3.28^	1.49 ± 0.17	= –3	N.T.	4.01 ± 0.06 ^‡ ‡^	4.24 ± 0.07 ^‡ ‡^	1.23 ± 0.01 ^‡ ‡^	Leach
L109A^3.29^	1.17 ± 0.14	= –3	N.T.	3.11 ± 0.09 ^‡ ‡^	4.28 ± 0.14 ^‡ ‡^	2.54 ± 0.10 ^‡ ‡^	Leach
D112E^3.32^	–0.80 ± 0.16 ^‡ ‡^	= –3	N.T.	<2	5.56 ± 0.13	0.39 ± 0.11 ^‡ ‡^	Leach
D112N^3.32^	N.D.	N.D.	N.T.	3.19 ± 0.02 ^‡ ‡^	5.79 ± 0.2 ^‡ ‡^	0.74 ± 0.08	Leach
Y113A^3.33^	N.T.	N.T.	N.T.	2.98 ± 0.12 ^‡ ‡^	4.97 ± 0.15	0.80 ± 0.10 ^‡ ‡^	Leach
S116A^3.36^	0.82 ± 0.17 ^‡ ‡^	–0.35 ± 0.45 ^‡ ‡^	N.T.	3.61 ± 0.10 ^‡ ‡^	5.12 ± 0.08	1.54 ± 0.05	Leach
N117A^3.37^	0.80 ± 0.27 ^‡ ‡^	–0.27 ± 0.16 ^‡ ‡^	N.T.	3.64 ± 0.04 ^‡ ‡^	5.30 ± 0.15	1.57 ± 0.13	Leach
V120A^3.40^	1.47 ± 0.11	1.20 ± 0.19	N.T.	5.63 ± 0.05 ^‡ ‡^	5.41 ± 0.10	1.83 ± 0.11	Leach
D129E^3.49^	1.45 ± 0.24	0.78 ± 0.16	N.T.	5.04 ± 0.07	5.59 ± 0.12	1.61 ± 0.16	Leach
D129N^3.49^	2.56 ± 0.39	1.86 ± 0.12	N.T.	5.54 ± 0.10 ^‡ ‡^	5.37 ± 0.20	1.86 ± 0.22	Leach
W164A^4.57^	N.D. (3)	= –3	2.17 ± 0.64***	3.95 ± 0.24	5.15 ± 0.28	ND	Current/Thal
F170A^4.63^	3.13 ± 0.17 (5)	2.66 ± 0.12*	3.58 ± 0.17*	4.77 ± 0.2	4.53 ± 0.06	2.23 ± 0.13	Current/Thal
W171A^4.64^	2.59 ± 0.17 (5)	1.31 ± 0.11	3.11 ± 0.13	3.91 ± 0.21	4.56 ± 0.15	2.00 ± 0.09	Current/Thal
Q172A^4.65^	3.05 ± 0.33 (5)	1.18 ± 0.31	2.71 ± 0.16	4.02 ± 0.09	4.99 ± 0.03	1.54 ± 0.08	Current/Thal
F173A^4.66^	3.39 ± 0.11(4)	2.03 ± 0.10*	3.31 ± 0.23*	4.09 ± 0.01	4.78 ± 0.19	1.90 ± 0.14	Current/Thal
Q184A^45.49^	3.01 ± 0.10 (4)	1.05 ± 0.08	2.08 ± 0.12	4.25 ± 0.12	5.36 ± 0.04	1.70 ± 0.05	Current/Thal
F186A^45.51^	1.99 ± 0.11	N.D.	N.T.	4.85 ± 0.06	NR	NR	Nawaratne
I187A^45.52^	2.57 ± 0.10	0.62 ± 0.09	2.07 ± 0.15	3.71 ± 0.12	5.46 ± 0.29	1.07 ± 0.29 ^‡ ‡^	Current/Thal
Q188A^45.53^	2.48 ± 0.15	0.99 ± 0.11	2.35 ± 0.16	4.6 ± 0.22	4.94 ± 0.08	1.49 ± 0.04	Current/Thal
F189A^45.54^	2.28 ± 0.11	1.25 ± 0.09	2.67 ± 0.11	4.65 ± 0.02	5.09 ± 0.13	1.99 ± 0.08	Current/Thal
L190A^45.55^	2.50 ± 0.14	1.32 ± 0.12	2.81 ± 0.12	4.20 ± 0.06	4.92 ± 0.1	2.06 ± 0.09	Current/Thal
W413A^6.48^	0.66 ± 0.12***,* (4)	0.61 ± 0.13***	3.54 ± 0.09***,*	3.47 ± 0.06 ^‡ ‡^	4.51 ± 0.37	2.45 ± 0.36	Current/Thal
Y416A^6.51^	N.T.	N.T.	N.T.	2.85 ± 0.10 ^‡ ‡^	NR	NR	Thal
N423A^6.58^	3.44 ± 0.15 (3)	0.82 ± 0.10	1.43 ± 0.19*	4.41 ± 0.15	5.02 ± 0.06	1.18 ± 0.08 ^‡ ‡^	Current/Thal
Q427A^6.62^	3.15 ± 0.14 (3)	0.99 ± 0.12	1.64 ± 0.12	4.46 ± 0.03	5.43 ± 0.06	1.36 ± 0.04	Current/Thal
S428P^6.63^	1.99 ± 0.09	1.40 ± 0.19	N.T.	5.14 ± 0.03 ^‡ ‡^	5.17 ± 0.15	1.81 ± 0.11	Nawaratne
D432N^7.32^	2.26 ± 0.12	1.25 ± 0.18	N.T.	5.19 ± 0.04 ^‡ ‡^	5.21 ± 0.2	1.37 ± 0.04	Nawaratne
W435A^7.35^	2.58 ± 0.17 (4)	= –3	N.R	3.37 ± 0.08 ^‡ ‡^	NR	NR	Current/Thal
Y439A^7.39^	0.60 ± 0.18 ^‡ ‡^	N.D.	N.T.	3.33 ± 0.10 ^‡ ‡^	5.84 ± 0.12	0.49 ± 0.03 ^‡ ‡^	Nawaratne
W440A^7.40^	3.69 ± 0.17^†††^(4)	0.84 ± 0.11	1.52 ± 0.20^†††^	4.29 ± 0.24	4.94 ± 0.06	0.96 ± 0.04 ^‡ ‡^	Current/Thal
C442A^7.42^	1.49 ± 0.16 ^‡ ‡^	0.82 ± 0.31	N.T.	4.04 ± 0.07 ^‡ ‡^	5.35 ± 0.06	1.81 ± 0.03	Nawaratne
Y443A^7.43^	0.50 ± 0.16 ^‡ ‡^	N.D.	N.T.	3.36 ± 0.01 ^‡ ‡^	6.22 ± 0.05 ^‡ ‡^	1.16 ± 0.01 ^‡ ‡^	Nawaratne

Values represent the mean ± SEM from three or more independent experiments with the number of individual experimental replicates from the current study shown in parenthesis.

N.T.: not tested; N.D.: not determined; N.R.: no response; ACh, acetylcholine.

* Data and analysis from pERK1/2 assays were generated in the current study, Nawaratne et al, J. Bio. Chem. 2010, and Leach et al, Mol. Pharm. 2011. logτ ACh were calculated from the operational model of agonism. log τ LY298 and log αβ were calculated using a simplified operational model of allosterism.

† Data and analysis from [^3^H]-QNB interaction binding assays were generated in Nawaratne et al, J. Bio. Chem. 2010, Leach et al, Mol. Pharm., and Thal et al, Nature 2016.

‡ logτ_C_ = logarithm of the operational efficacy parameter corrected for receptor expression using the maximum number of receptor binding sites as previously determined from Nawaratne et al, J. Bio. Chem. 2010, Leach et al, Mol. Pharm., and Thal et al, Nature 2016.

§ Logarithm of the functional cooperativity factor between ACh and LY298.

¶ Negative logarithm of the orthosteric (pK_i_) or allosteric (pK_B_) equilibrium dissociation constant.

** Logarithm of the binding cooperativity factor between ACh and LY298.

†† Values of logτ_C_ ACh, log τ_C_ LY298, and log αβ that were calculated in this study. Other parameters are from Thal et al., 2016.

‡ ‡ Values are significantly different from WT M_4_ mAChR as determined in previous studies.

§ § All values are from Nawaratne et al, *J. Bio. Chem*. 2010 with logτ corrected for receptor expression.

¶ ¶ All values are from Leach et al, *Mol. Pharm*. 2011.

*** Parameters determined from the full Operational Model of Allosterism.

††† Values are significantly different from WT M_4_ mAChR (p<0.05) calculated by a one-way ANOVA with a Dunnett’s post-hoc test.

Mutation of residues that directly surround ACh primarily decreased the efficacy of ACh (Figure 6D, Table 5). One exception was W98^23.50^ (an ECL1 residue numbered 23.X denoting its position between TM2 and TM3 with X.50 denoting the most conserved residue), a residue that was recently identified in a deep scanning mutagenesis study as a conserved class A residue that is intolerant to mutation (Jones et al., 2020) and stabilizes the conserved disulfide bridge between ECL1 and TM3 that is important for the stability of the active state of many GPCRs including mAChRs (Hulme, 2013). Interestingly, residues that affect the efficacy of LY298 include nearly all of the residues that also affect ACh efficacy, along with residues that link to the allosteric site and surround the LY298 binding site (Figure 6E, Table 5). This suggests that the direct signaling of LY298 via the allosteric site is nonetheless linked through a similar network of residues and requires a functional orthosteric site for the transduction of signaling, and that mechanism involves equivalent closure of the orthosteric binding site, consistent with the thermodynamic reciprocity of cooperativity (Canals et al., 2011).

Residues Y89^2.61^, N432^6.58^, W435^7.35^, and W440^7.40^ were identified as residues that, when mutated to alanine, significantly decreased the functional modulation between ACh and LY298 (Figure 6F, Table 5). In prior work, all four residues were also shown to contribute to LY298 binding or affinity modulation (Thal et al., 2016). Surprisingly, three mutations resulted in increased functional modulation by LY298. Of particular interest was, again, the rotamer toggle switch residue W413^6.48^. Mutation of W413^6.48^ to alanine significantly impaired the efficacy of ACh but only reduced the efficacy of LY298 by twofold, such that ACh and LY298 had similar efficacy for this mutant (Figure 6G–I, Table 5). Interestingly, the functional modulation (αβ) between ACh and LY298 increased to over 3600 (a 20-fold increase vs. WT) at W413A^6.48^. Similar results were observed in the TruPath assay with ACh, Ipx, and LY298 (Figure 6—figure supplement 2 [mutant], Figure 1—figure supplement 1B [WT]). However, with VU154, the functional modulation was considerably reduced with ACh and non-existent with Ipx, in line with our TruPath experiments at the WT M_4_ mAChR. These results show that, at the M_4_ mAChR, the rotamer toggle switch residue is important for the signaling efficacy of orthosteric agonists and PAM-agonists but does not impair the process of functional allosteric modulation. Thus, suggesting that the stability of LY298 co-binding with agonists can restore impaired function, while the less stable binding of VU154 does not. Together with the observation that most of the structural differences between the active-state M_4_ mAChR structures occur at or above W413^6.48^, we propose that this residue has a strong role in maintaining the conformational dynamics of the receptor and is a key trigger for robust signal transduction.

### A molecular basis of species selectivity

One of the main advantages of allosteric modulators is the ability to selectivity target highly conserved proteins. The mAChRs are the prime example where allosteric modulators have been designed to selectively target specific subtypes. To date, the only PAM-bound mAChR structures are ones with LY2119620, a PAM that has activity at both the M_2_ and M_4_ mAChRs. Similarly, LY298 has activity at the M_2_ mAChR. However, the allosteric properties of VU154 are differentially affected by the species of the receptor (Wood et al., 2017b; Wood et al., 2017a). At the human M_4_ mAChR, LY298 displays robust binding affinity modulation, functional modulation, and allosteric agonism, while VU154 has comparatively weaker allosteric properties (Figure 1, Table 1). Conversely, at the mouse M_4_ mAChR, VU154 has a high degree of positive binding modulation, functional modulation, and allosteric agonism that is comparable to LY298 at the human M_4_ mAChR (Figure 7—figure supplements 1 and 2, Table 1). Therefore, we aimed to determine whether our prior findings could be used to explain the selectivity of VU154 between the human and mouse receptors.

**Figure 7.**
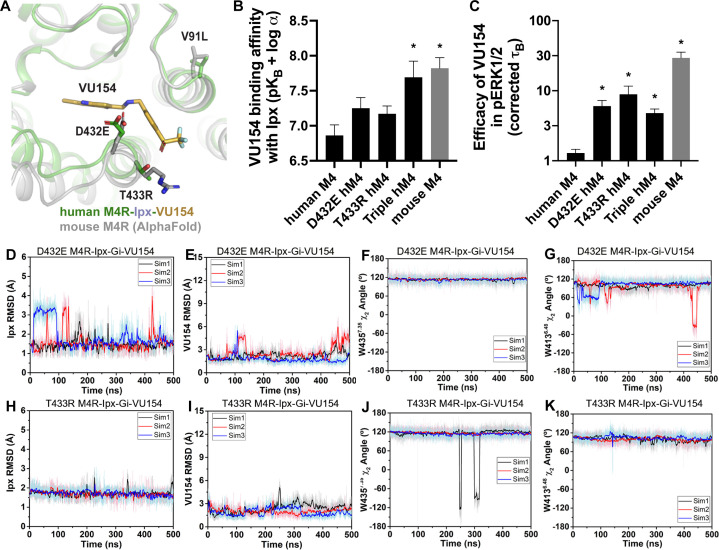
A molecular mechanism for the species selectivity for VU154. (**A**) Comparison of the cryo-electron microscopy (cryo-EM) structure of the human M4 muscarinic acetylcholine receptor (mAChR) bound to Ipx-VU154 with the AlphaFold model of the mouse M4 mAChR (Jumper et al., 2021; Varadi et al., 2022). The three residues that differ between species and within the core 7TM bundle from the human receptor (V91, D432, and T433) are shown as sticks along with the corresponding residues from the mouse receptor. (**B**) The binding affinity of VU154 for the Ipx-bound conformation (pK_B-Ipx_ = pK_B_ + α) determined from [^3^H]-NMS binding experiments. Values calculated with data from Figure 7—figure supplement 1 with propagated error. (**C**) Efficacy of VU154 (τ_B_ – corrected for receptor expression) of pERK1/2 signaling from data in Figure 7—figure supplement 2. (**D–K**) Time courses of obtained from Gaussian accelerated molecular dynamics (GaMD) simulations of the (**D–G**) D432E and (**H–K**) T433R mutant M_4_R-Ipx-G_i1_-VU154 systems with (**D, H**) Ipx RMSDs, (**E, I**), VU154 root mean square deviations (RMSDs), (**F, J**) W435^7.35^ χ_2_ angle, and (**G, K**) W413^6.48^ χ_2_ angle. Data shown are mean ± SEM from three or more experiments performed in duplicate with the pharmacological parameters determined from a global fit of the data. *Indicates statistical significance (p<0.05) relative to WT as determined by a one-way ANOVA with a Dunnett’s post-hoc test. Figure 7—source data 1.Related to Figure 7.

The amino acid sequences of the human and mouse M_4_ mAChRs are highly conserved, with most of the differences occurring between the long third intracellular loop and the N- and C- termini. As shown in Figure 7A, only three residues differ between the human and mouse M_4_ mAChR with respect to the transmembrane domain. Specifically, residue V91 (L in mouse) at the top of TM2 points into the lipid bilayer, and D432 and T433 (E and R in mouse), which are located at the top of TM7 and form part of the allosteric binding site near VU154.

**Figure 7—figure supplement 1.**
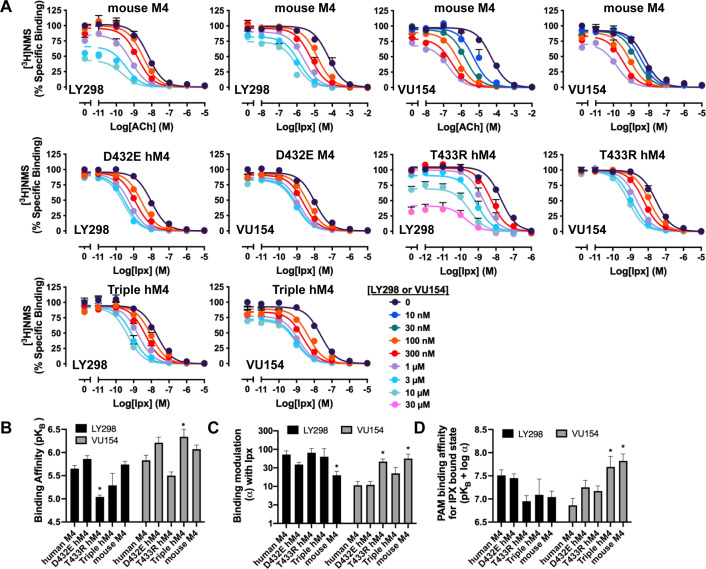
Binding parameters of positive allosteric modulators (PAMs) at the human and mouse M_4_ muscarinic acetylcholine receptors (mAChRs). (**A**) Concentration–response curves of the orthosteric and allosteric ligands in [^3^H]-NMS binding assays at the mouse M_4_ mAChR, D432E, T433R, and the V91L, D432E, T433R triple mutant of the human M_4_ mAChR. (**B–D**) Quantification of data from (**A**) to calculate (**B**) equilibrium binding affinities (pK_B_) of the PAMs, (**C**) the degree of binding modulation (α) between iperoxo (Ipx) and PAMs, and the modified affinities (**D**) α/K_B_. See Table 1. All data are mean ± SEM of three or more independent experiments performed in duplicate or triplicate with the pharmacological parameters determined from a global fit of the data. The error in (**D**) was propagated using the square root of the sum of the squares. *Indicates statistical significance (p<0.05) relative to WT as determined by a one-way ANOVA with a Dunnett’s post-hoc test. Figure 7—figure supplement 1—source data 1.Related to Figure 7—figure supplement 1.

**Figure 7—figure supplement 2.**
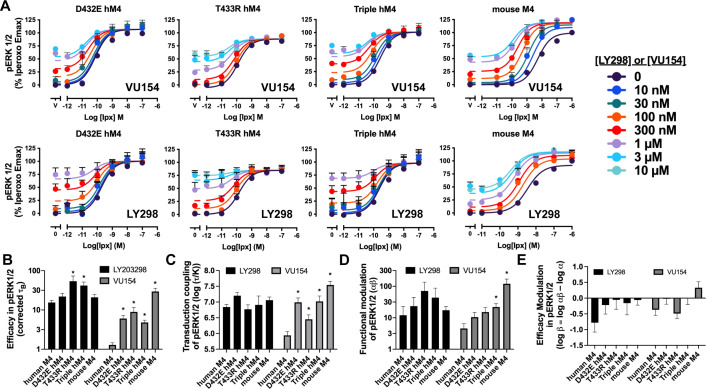
Functional parameters of the positive allosteric modulators (PAMs) at the human and mouse M_4_ muscarinic acetylcholine receptors (mAChRs) in pERK1/2 signaling assays. (**A**) Concentration–response curves of an interaction between iperoxo (Ipx) and the PAMS VU154 and LY298 in pERK1/2 at the mouse M_4_ mAChR, D432E, T433R, and the V91L, D432E, T433R triple mutant of the human M_4_ mAChR. (**B–E**) Quantification of data from (**A**) to calculate (**B**) the signaling efficacy (τ_A_ and τ_B_) and (**C**) the transduction coupling coefficients (log (τ/K)) of each ligand, (**D**) the functional cooperativity (αβ) between ligands, and (**E**) the efficacy modulation (β) between ligands. See Table 1. Figure 7—figure supplement 2—source data 1.Related to Figure 7—figure supplement 2.

**Figure 7—figure supplement 3.**
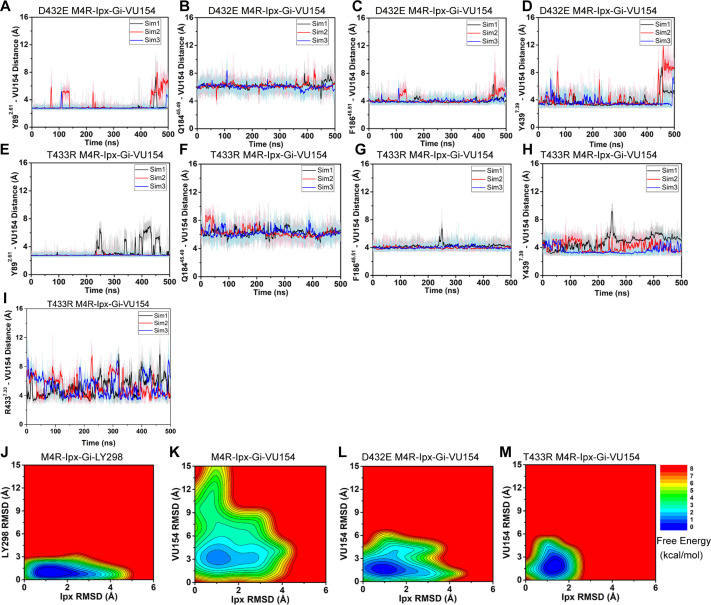
Gaussian accelerated molecular dynamics (GaMD) simulations of D432E and T433R human M_4_ muscarinic acetylcholine receptor (mAChR) mutants. (**A–H**) Time courses obtained from GaMD simulations of the (**A–D**) D432E and (**E–H**) T433R mutant M_4_R-Ipx-G_i1_-VU154 systems with (**A, E**) Y89^2.61^ – VU154 distance, (**B, F**) Q184^45.49^ – VU154 distance, (**C, G**) F186^45.51^ – VU154 distance, and (**D, H**) Y439^7.39^ – VU154 distance. with residues (**A, E**) Y89^7.39^, (**B, F**) F186^45.51^, (**C, G**) Y439^7.39^, and (**D, H**) Q184^45.49^. (**I**) Distance between R433^7.33^ to the sulfoxide group of VU154 from GaMD simulations of the T433R M_4_R-Ipx-G_i1_-VU154 mutant. (**J–M**) 2D free energy profile of the root mean square deviations (RMSDs) of LY298 and VU154 with Ipx. See Table 3.

Previous work suggested that residues D432 and T433 were important for differences in the species selectivity of LY298 (Chan et al., 2008). As such, we examined two single D432E and T433R mutants and a V91L/D432E/T433R triple mutant of the human receptor, along with the mouse M_4_ mAChR in radioligand binding and pERK1/2 experiments using Ipx and both PAMs (Figure 7—figure supplements 1 and 2, Table 1). For LY298, there were no statistically significant differences in binding or function between species and across the mutants that were more than threefold in effect. In contrast, VU154 had a tenfold higher binding affinity for the Ipx-bound mouse M_4_ mAChR (compare Figure 1G with Figure 7B). The affinity of VU154 increased by 2.5-fold at the D432E and T433R mutants and the triple mutant matched the affinity of the mouse receptor (Figure 7B). In functional assays, similar results were observed for VU154 with Ipx at the mouse M_4_ mAChR, with significant increases in the efficacy (τ_B_ – corrected for receptor expression), transduction coefficients (τ_B_/K_B_), and functional modulation (αβ) (Figure 7B, Figure 7—figure supplements 1 and 2, Table 1). Relative to the WT M_4_ mAChR, the efficacy (Figure 7C), transduction coefficients, and functional modulation of VU154 increased for all of the mutants (Figure 7—figure supplements 1 and 2, Table 1); however, none of the values fully matched the mouse receptor. Nevertheless, these results indicate that V91L, D432E, and T433R play a key role in mediating the species selectivity of VU154.

Our prior findings suggest the robust allosteric activity of LY298 at the human M_4_ mAChR was due to stable interactions with the receptor. As a proof-of-principle, we questioned whether GaMD simulations would produce a stable binding mode for VU154 with D432E and T433R mutations to the VU154-Ipx-bound M_4_R-G_i1_ cryo-EM structure that was similar to our previously observed stable binding pose of LY298 (Figure 4). Excitingly, both the D432E and T433R mutants resulted in a dynamic profile of VU154 that matched our GaMD simulations of LY298 from the LY298-Ipx-bound M_4_R-G_i1_ cryo-EM structure, including stabilized VU154 binding, constrained χ_2_ rotamer conformations of W435^7.35^ and W413^6.48^, and stable binding interactions with Y89^2.61^, Y439^7.39^, Q184^45.49^, and F186^45.51^ (Figure 7D–K, Figure 7—figure supplement 3, Videos 9 and 10). The GaMD simulations also suggest that a potential interaction between the mutant residue T433R and the sulfoxide group of VU154 was more stable (5.2 ± 1.5 Å; Figure 7—figure supplement 3I) versus the WT residue T433 (6.56 ± 2.1 Å, Figure 4—figure supplement 1J), albeit the distance of this interaction was far apart and would be better validated by structure determination of VU154 with the mouse M_4_ mAChR.

**Video 9.** Movie from one VU154-Ipx-M_4_R(D432E)-G_i1_ Gaussian accelerated molecular dynamics (GaMD) simulation.

**Video 10.** Movie from one VU154-Ipx-M4R(T433R)-G_i1_ Gaussian accelerated molecular dynamics (GaMD) simulation.

Collectively, these findings reiterate the importance of receptor dynamics in the determination of allosteric modulator selectivity as even subtle differences in amino acid residues between species may result in profound changes in overall stability of the same PAM-agonist-receptor complex.

## Discussion

**Figure 8.**
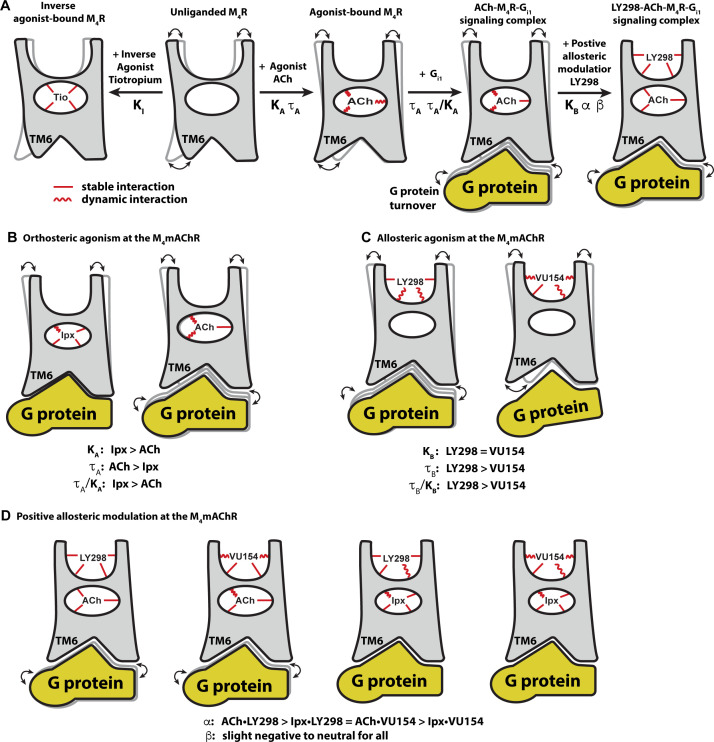
Conformational dynamics of the allostery at M_4_ muscarinic acetylcholine receptor (mAChR) signaling complexes. (**A**) A schematic cartoon illustrating the conformational states of the ligands and the M_4_ mAChR when bound to different types of ligands and transducer, along with the resulting dynamic profiles. Pharmacological parameters related to each conformational change are shown. Stable ligand–receptor interactions are denoted by a straight line and less-stable (more dynamic) interactions are denoted by a wavy line. (**B**) Iperoxo (Ipx) bound the M_4_ mAChR with a higher affinity and more stability than ACh but had lower efficacy. ACh being more loosely bound and coupled to G protein may facilitate more G protein turnover accounting for its higher efficacy. (**C**) LY298 and VU154 bound to the M_4_ mAChR with similar affinity for the receptor, but LY298 was found to bind more stably. LY298 had a higher efficacy than VU154, suggesting that allosteric agonism at the M_4_ mAChR is mediated by stabilization of the extracellular vestibule (ECV). (**D**) The positive allosteric modulators (PAMs) LY298 and VU154 display robust binding modulation at the M_4_ mAChR with LY298 having a stronger allosteric effect. Both PAMs displayed stronger binding modulation with the agonist ACh versus Ipx, an example of probe dependence. Both PAMs also displayed a slight negative to neutral effect on the efficacy of the agonists, suggesting that their mechanism of action is largely through binding.

Major advances have been made in recent years in the appreciation of the role of GPCR allostery and its relevance to modern drug discovery (Changeux and Christopoulos, 2016; Wootten et al., 2013). Despite an increase in the number of reported high-resolution GPCR structures bound to allosteric ligands (Thal et al., 2018), there remains a paucity of molecular-level details about the interplay between the complex chemical and pharmacological parameters that define allostery at GPCRs. By combining detailed pharmacology studies, multiple high-resolution cryo-EM structures of the M_4_ mAChR bound to two pharmacologically different agonists and PAMs, and GaMD simulations, we have now provided exquisite in-depth insights into the relationship between both structure and dynamics that govern multiple facets of GPCR allostery (Figure 8A).

Comparison of the ACh- and Ipx-bound M_4_ mAChR structures revealed that Ipx bound in a smaller binding pocket (Figure 3G and H), and GaMD simulations showed that Ipx formed more stable interactions with the receptor (Figure 3—figure supplement 1). These observations likely explained why Ipx exhibited greater than 1000-fold higher binding affinity than ACh (Figure 1D), being consistent with studies of other agonists at the β_1_-adrenoceptor and the M_1_ mAChR (Brown et al., 2021; Warne et al., 2019; Figure 8B). The observation that ACh was a more efficacious agonist than Ipx (Table 1) yet bound with lower affinity and less stable interactions than Ipx was paradoxical. Kenakin and Onaran, 2002 previously opined on the paradox between ligand binding affinity and efficacy and showed via simulations that, in general, there was a negative correlation between binding affinity and efficacy. One interpretation of these results was that the ACh-bound M_4_ mAChR more readily sampled receptor conformations that engaged with the transducers (Manglik et al., 2015). Similarly, the ACh-bound M_4_ mAChR may also have faster G protein turnover than Ipx due to Ipx-M_4_R-G_i1_ forming a more stable ternary complex (Furness et al., 2016; Figure 8B).

It is worth noting that structures of GPCRs bound to agonists with different pharmacological properties (full, partial, and biased agonists) have now been reported for some GPCRs (Liang et al., 2018a; Masureel et al., 2018; McCorvy et al., 2018; Ring et al., 2013; Wacker et al., 2013; Warne et al., 2012; Wingler et al., 2019). However, insights gained from such cryo-EM and X-ray crystallography structures may be limited due to the role that the bound transducer plays on the observed final receptor conformation, and not necessarily due solely to the properties of the ligand. The ultimate underlying conformational differences, therefore, are likely to be subtle and dynamic (Seyedabadi et al., 2022), requiring application of additional techniques such as NMR spectroscopy, single-molecule FRET and MD simulations for furthering our understanding (Cao et al., 2021; Cong et al., 2021; Gregorio et al., 2017; Huang et al., 2021; Katayama et al., 2021; Liu et al., 2012b; Solt et al., 2017; Sušac et al., 2018; Xu et al., 2023; Ye et al., 2016).

Indeed, if considering this issue from the perspective of allosteric modulators of GPCRs, our study highlights that two PAMs with distinctly different pharmacological profiles (Figure 1) may bind to and stabilize receptor conformations that were very similar when viewed as static structures (Figure 4). Yet, in contrast, the 3DVA analysis from our cryo-EM structures suggested differences in the dynamics of the cryo-EM structures that were explored further in GaMD simulations (Figure 4C) and revealed that LY298 had a more stable binding pose and interactions with the receptor than VU154 in the PAM-agonist–receptor–transducer-bound conformation. These observations were consistent with LY298 having greater positive binding cooperativity than VU154 (Figure 1E) and suggest that GaMD simulations of GPCRs bound to allosteric ligands could be an extremely valuable tool for drug discovery and optimization (Bhattarai and Miao, 2018).

Pharmacological analysis revealed that LY298 is a better PAM-agonist than VU154 with respect to efficacy (Figure 1H) in the G_i1_ TruPath and pERK1/2 signaling assays (Figure 1—figure supplement 2B). GaMD simulations of the PAM–receptor–transducer and PAM–receptor bound complexes, again showed that LY298 more stably interacted with the receptor (Figure 4) and in the absence of G protein better stabilized the duration of the active conformation of the receptor (Figure 5). These findings were not contradictory to our above findings that ACh was more efficacious than Ipx despite having weaker interactions with the receptor because when the affinity of the ligands was accounted for in the transduction coupling coefficients, the rank order was Ipx >> ACh ~ LY298 > VU154 (Figure 1I). Furthermore, these results were in accordance with the observations of Kenakin and Onaran that ligands with the same binding affinity can also have differing efficacies (and vice versa). In addition, the mechanism of agonism for allosteric ligands that bind to the ECV may differ (Xu et al., 2021). Prior work by DeVree et al., 2016 established that allosteric coupling of G proteins to the unliganded active receptor conformation promoted closure of the ECV region. This allosteric coupling is reciprocal and stabilizing the ECV region by PAMs likely leads to increased efficacy (Figure 8).

The PAMs, LY298 and VU154, also displayed stronger allosteric effects with ACh than with Ipx, an observation known as probe dependence (Figure 1E–G). Probe dependence can have substantial implications on how allosteric ligands are detected, validated, and their potential therapeutic utility (Kenakin, 2005). Examples of probe dependence are not limited to studies on mAChRs and have been observed across multiple receptor families (Christopoulos, 2014; Gentry et al., 2015; Pani et al., 2021; Slosky et al., 2020; Wang et al., 2021b). GaMD simulations comparing the PAMs co-bound with either Ipx or ACh showed that the PAMs had a stabilizing effect on ACh, whereas the stability of Ipx was slightly reduced by the PAMs likely because the binding of Ipx was already stable. This is a sensible explanation from thermodynamic principles. Another explanation invokes the two-state receptor model (Canals et al., 2011), which stipulates that the degree of positive modulation for PAMs increases with an increase in the efficacy of the agonists. The pharmacology data support this model as ACh was more efficacious than Ipx and was better modulated by both PAMs (Figure 8D). These observations are also consistent with recent studies that suggest that conformational dynamics between agonist and receptor are important for functional signaling (Bumbak et al., 2020; Cary et al., 2022; Deganutti et al., 2022; O’Connor et al., 2015).

The findings presented here provide new insights into the allosteric signaling and allosteric modulation of GPCRs by combining the analytical analysis of multiple pharmacology assays with cryo-EM structures and GaMD simulations. Overall, these results provide a framework for future mechanistic studies and, ultimately, can aid in the discovery, design, and optimization of allosteric drugs as novel therapeutic candidates for clinical progression.

### Limitations of the study

The complexities of GPCR signaling cannot be fully explained by any single receptor or set of experiments. This study was limited to the investigation of two agonists and two PAMs at the human M_4_ mAChR. Future studies will be required to determine how these results extrapolate to other classes of ligand, mAChR subtypes, and GPCRs. For instance, this study determined the structures of the M_4_ mAChR bound with the ligands ACh, Ipx, Ipx-LY298, and Ipx-VU154. It is possible that structures of the M_4_ mAChR bound with ACh-LY298 and ACh-VU154 could reveal different receptor conformations (although GaMD simulations already performed on their docked complexes and the conformational differences between the Ipx-bound cryo-EM structures suggest otherwise). Similarly, structures of the M_4_ mAChR bound in complex with either PAM alone may provide better insights into direct allosteric agonism. However, we note that our attempt at determining an LY298-bound complex did not have sufficient stability for the determination of a high-resolution structure, as also supported by our GaMD simulations. Additionally, our cryo-EM structures and MD-simulations utilized an M_4_ mAChR sequence with a large portion of the third intracellular loop removed and were complexed with a dominant negative mutant of Gα_i1_ and stabilized with the antibody scFv16. This contrasts with our pharmacological characterization of the ligands that were performed on the WT M_4_ mAChR. Further investigation into the molecular determinants of species selectivity is also warranted, as is the need for future experiments that incorporate the combined interplay between dynamics/kinetics of ligands, receptor, transducer recruitment and activation.

## Materials and methods

### Bacterial strains

DH5α (New England Biolabs) and DH10bac (Thermo Fisher Scientific) *Escherichia coli* cells were grown in LB at 37°C.

### Cell culture

Tni and Sf9 cells (Expression Systems) were maintained in ESF-921 media (Expression Systems) at 27°C. Flp-In Chinese hamster ovary (CHO) (Thermo Fisher Scientific) cells stably expressing human M_4_ mAChR or mutant constructs were maintained in Dulbecco’s modified Eagle’s medium (DMEM, Invitrogen) containing 5% fetal bovine serum (FBS; ThermoTrace) and 0.6 μg/ml of Hygromycin (Roche) in a humidified incubator (37°C, 5% CO_2_, 95% O_2_). HEK293A cells were grown in DMEM supplemented with 5% FBS at 37°C in 5% CO_2_. Cell lines were authenticated by vendor and confirmed negative for mycoplasma contamination using the Lonza MycoAlert Mycoplasma Detection Kit (#LT07-318).

### Radioligand binding assays

Flp-In CHO cells stably expressing M_4_ mAChR constructs were seeded at 10,000 cells/well in 96-well white clear bottom isoplates (Greiner Bio-one) and allowed to adhere overnight at 37°C, 5% CO_2_, and 95% O_2_. Saturation binding assay was performed to quantify the receptor expression and equilibrium dissociation constant of the radioligand [^3^H]-NMS (PerkinElmer, specific activity 80 Ci/mmol). Briefly, plates were washed once with phosphate-buffered saline (PBS) and incubated overnight at room temperature (RT) with 0.01–10 nM [^3^H]-NMS in Hanks’s balanced salt solution (HBSS)/10 mM HEPES (pH 7.4) in a final volume of 100 μl. For binding interaction assays, cells were incubated overnight at RT with a specific concentration of [^3^H]-NMS (pK_D_ determined at each receptor in saturation binding) and various concentrations of ACh or Ipx in the absence or presence of increasing concentrations of each allosteric modulator. In all cases, nonspecific binding was determined by the coaddition of 10 μM atropine (Sigma). The following day, the assays were terminated by washing the plates twice with ice-cold 0.9% NaCl to remove the unbound radioligand. Cells were solubilized in 100 μl per well of Ultima Gold (PerkinElmer), and radioactivity was measured with a MicroBeta plate reader (PerkinElmer).

### G protein activation assay

Upon 60–80% confluence, HEK293A cells were transfected transiently using polyethylenimine (PEI, Polysciences) and 10 ng per well of each of pcDNA3.1-hM4 mAChR (WT or mutant), pcDNA5/FRT/TO-Gα_i1_-RLuc8, pcDNA3.1-β_3_, and pcDNA3.1-Gγ_9_-GFP2 at a ratio of 1:1:1:1 ratio with 40 ng of total DNA per well. Cells were plated at 30,000 cells per well into 96-well Greiner CELLSTAR white-walled plates (Sigma-Aldrich). 48 hr later, cells were washed with 200 μl phosphate buffer saline (PBS) and replaced with 70 μL of 1× HBSS with 10 mM HEPES. Cells were incubated for 30 min at 37°C before addition of 10 μl of 1.3 μM Prolume Purple coelenterazine (Nanolight Technology). Cells were further incubated for 10 min at 37C° before BRET measurements were performed on a PHERAstar plate reader (BMG Labtech) using 410/80 nm and 515/30 nm filters. Baseline measurements were taken for 8 min before addition of drugs or vehicle to give a final assay volume of 100 μl and further reading for 30 min. BRET signal was calculated as the ratio of 515/30 nm emission over 410/80 nm emission. The ratio was vehicle corrected using the initial 8 min of baseline measurements and then baseline corrected again using the vehicle-treated wells. Data were normalized using the maximum agonist response to allow for grouping of results using an area under the curve analysis in Prism. Data were analyzed at timepoints of 4, 10, and 30 min yielding similar results.

### Phospho-ERK1/2 assay

The level of phosphorylated extracellular signal-regulated protein kinase 1/2 (pERK1/2) was detected using the AlphaScreen SureFire Kit (PerkinElmer Life and Analytical Sciences). Briefly, FlpIn CHO cells stably expressing the receptor were seeded into transparent 96-well plates at a density of 20,000 cells/well and grown overnight at 37°C, 5% CO_2_. Cells were washed with PBS and incubated in serum-free DMEM at 37°C for 4 hr to allow FBS-stimulated pERK1/2 levels to subside. Cells were stimulated with increasing concentrations of ACh or Ipx in the absence or presence of increasing concentrations of the allosteric modulator at 37°C for 5 min (the time required to maximally promote ERK phosphorylation for each ligand at each M_4_ mAChR construct in the initial time-course study; data not shown). For all experiments, stimulation with 10% (*v/v*) FBS for 5 min was used as a positive control. The reaction was terminated by the removal of media and lysis of cells with 50 μl of the SureFire lysis buffer (TGR Biosciences). Plates were then agitated for 5 min and 5 μl of the cell lysate was transferred to a white 384-well ProxiPlate (Greiner Bio-one) followed by the addition of 5 μl of the detection buffer (a mixture of activation buffer:reaction buffer:acceptor beads:donor beads at a ratio of 50:200:1:1). Plates were incubated in the dark for 1 hr at 37°C followed by measurement of fluorescence using an Envision plate reader (PerkinElmer) with standard AlphaScreen settings. Data were normalized to the maximal response mediated by 10 μM ACh, Ipx, or 10% FBS.

### Purification of scFv16

Tni insect cells were infected with scFv16 baculovirus at a density of 4 million cells per ml and harvested at 60 hr post infection by centrifugation for 10 min at 10,000 × *g*. The supernatant was pH balanced to pH 7.5 by the addition of Tris pH 7.5, and 5 mM CaCl_2_ was added to quench any chelating agents, then left to stir for 1.5 hr at RT. The supernatant was then centrifuged at 30,000 × *g* for 15 min to remove any precipitates. 5 ml of EDTA-resistant Ni resin (Cytivia) was added and incubated for 2 hr at 4^o^C while stirring. Resin was collected in a glass column and washed with 20 column volumes (CVs) of high salt buffer (20 mM HEPES pH 7.5, 500 mM NaCl, 20 mM imidazole) followed by 20 CVs of low salt buffer (20 mM HEPES pH 7.5, 100 mM NaCl, 20 mM imidazole). Protein was then eluted using 8 CV of elution buffer (20 mM HEPES pH 7.5, 100 mM NaCl, 250 mM imidazole) until no more protein was detected using Bradford reagent (Bio-Rad Laboratories). Protein was concentrated using a 10 kDa Amicon filter device (Millipore) and aliquoted into 1 mg aliquots for further use.

### Expression and purification of M_4_R-G_i1_-scFv16 complexes

The human M_4_ mAChR with residues 242–387 of the third intracellular loop removed and the N-terminal glycosylation sites (N3, N9, N13) mutated to D was expressed in Sf9 insect cells, and human DNG_αi1_ and His6-tagged human G_β1γ2_ were co-expressed in Tni insect cells. Cell cultures were grown to a density of 4 million cell per ml for Sf9 cells and 3.6 million per ml for Tni cells and then infected with either M_4_ mAChR baculovirus or both G_αi1_ and G_β1γ2_ baculovirus, at a ratio of 1:1. M_4_ mAChR expression was supplemented with 10 mM atropine. Cultures were grown at 27°C and harvested by centrifugation 60–72 hr (48 hr for Hi5 cells) post infection. Cells were frozen and stored at –80°C for later use. 1–2 l of the frozen cells were used for each purification.

Cells expressing M_4_ mAChR were thawed at RT and then dounced in the solubilization buffer containing 20 mM HEPES pH 7.5, 10% glycerol, 750 mM NaCl, 5 mM MgCl_2_, 5 mM CaCl_2_, 0.5% LMNG, 0.02% CHS, 10 µM atropine, and cOmplete Protease Inhibitor Cocktail (Roche) until homogeneous. The receptor was solubilized for 2 hr at 4°C while stirring. The insoluble material was removed by centrifugation at 30,000 × *g* for 30 min followed by filtering the supernatant and batch-binding immobilization to M1 anti-flag affinity resin, previously equilibrated with high salt buffer, for 1 hr at RT. The resin with immobilized receptor was then washed using a peristaltic pump for 30 min at 2 ml/min with high salt buffer: 20 mM HEPES pH 7.5, 750 mM NaCl, 5 mM MgCl_2_, 5 mM CaCl_2_, 0.5% lauryl maltose neopentyl glycol (LMNG, Anatrace), 0.02% cholesterol hemisuccinate (CHS, Anatrace) followed by low salt buffer: 20 mM HEPES pH 7.5, 100 mM NaCl, 5 mM MgCl_2_, 5 mM CaCl_2_, 0.5% LMNG, 0.02% CHS, and an agonist (5 µM Ipx, 1 µM Ipx with 10 µM VU154, or 100 µM ACh). While the receptor was immobilized on anti-FLAG resin, the DNGα_i1_ cell pellet was thawed, dounced, and solubilized in the solubilization buffer containing 20 mM HEPES pH 7.5, 100 mM NaCl, 5 mM MgCl_2_, 5 mM CaCl_2_, 0.5% LMNG, 0.02% CHS, apyrase (five units), and cOmplete Protease Inhibitor Cocktail. DNGα_i1_ was solubilized for 2 hr at 4°C followed by the centrifugation at 30,000 × *g* for 30 min to remove the insoluble material. Supernatant was filtered through a glass fiber filter (Millipore) and then added to the receptor bound to anti-Flag resin. Apyrase (five units), scFv16, and agonist (either 1 µM Ipx, 1 µM Ipx with 10 µM VU154, or 100 µM ACh) were added and incubated for 1 hr at RT with gentle mixing. The anti-FLAG resin was then loaded onto a glass column and washed with approximately 20 CVs of washing buffer: 20 mM HEPES pH 7.4, 100 mM NaCl, 5 mM MgCl_2_, 5 mM CaCl_2_, 0.01% LMNG, 0.001% CHS, agonist (1 µM Ipx, 1 µM Ipx with 10 µM VU154, or 100 µM ACh). Complex was eluted with size-exclusion chromatography (SEC) buffer: 20 mM HEPES pH 7.5, 100 mM NaCl, 5 mM MgCl_2_, 0.01% LMNG, 0.001% CHS and agonist (1 µM Ipx, or 1 µM Ipx with 10 µM VU154, or 100 µM ACh) with the addition of 10 mM EGTA and 0.1 mg/mL FLAG peptide. After the elution, an additional 1–2 mg of scFv16 was added and shortly incubated on ice before concentrating using a 100 kDa Amicon filter to a final volume of 500 µl. The sample was filtered using a 0.22 µm filter followed by SEC using a Superdex 200 increase 10/300 column (Cytivia) using SEC buffer. For the ACh- and VU154-Ipx-bound samples, the fractions containing protein were concentrated again and re-run over SEC using a buffer with half the amount of detergent in order to remove empty micelles. Samples were concentrated and flash frozen using liquid nitrogen. In case of the LY298-Ipx-bound sample, the sample was purified with 1 µM Ipx only. After SEC, the sample was then split in half, where one half was incubated with approximately 1.6 µM LY298 at 4°C overnight, and then concentrated and flash frozen in liquid nitrogen.

### EM sample preparation and data acquisition

Samples (3 µl) were applied to glow-discharged Quantifoil R1.2/1.3 Cu/Rh 200 mesh grids (Quantifoil) (M4R-G_i1_-Ipx and M4R-G_i1_-Ipx-LY298) or UltrAuFoil R1.2/1.3 Au 300 mesh grids (Quantifoil) (M4R-G_i1_-Ipx-VU154 and M4R-G_i1_-Ach) and were vitrified on a Vitrobot Mark IV (Thermo Fisher Scientific) set to 4°C and 100% humidity and 10 s blot time. Data were collected on a Titan Krios G3i 300 kV electron microscope (Thermo Fisher Scientific) equipped with GIF Quantum energy filter and K3 detector (Gatan). Data acquisition was performed in EFTEM NanoProbe mode with a 50 µM C2 aperture at an indicated magnification of ×105,000 with zero-loss slit width of 25 eV. The data were collected automatically with homemade scripts for SerialEM performing a nine-hole beam-image shift acquisition scheme with one exposure in the center of each hole. Experimental parameters specific to each collected data set is listed in Table 2.

### Image processing

Specific details for the processing of each cryo-EM data set are shown in Figure 2—figure supplement 2. Image frames for each movie were motion corrected using MotionCor2 (Zheng et al., 2017) and contrast transfer function (CTF)-estimated using GCTF (Zhang, 2016). Particles were picked from corrected micrographs using crYOLO (Wagner et al., 2019) or RELION-3.1 software Zivanov et al., 2018 followed by reference-free 2D and 3D classifications. Particles within bad classes were removed and remaining particles subjected to further analysis. Resulting particles were subjected to Bayesian polishing, CTF refinement, 3D auto-refinement in RELION, followed by another round of 3D classification and 3D refinement that yielded the final maps (Zivanov et al., 2018). Local resolution was determined from RELION using half-reconstructions as input maps. Due to the high degree of conformational flexibility between the receptor and G protein, a further local refinement was performed in cryoSPARC for the ACh-bound M_4_R-complex. A receptor-focused map was generated (2.75 Å), which was used to generate a PDB model of the ACh-bound M_4_R.

### Model building and refinement

An initial M_4_R template model was generated from our prior modeling studies of the M_4_ mAChR that was based on an active state M_2_ mAChR structure (PBD: 4MQT) (Kruse et al., 2013). An initial model for dominant negative Gα_i1_Gβ_1_Gγ_2_ was from a structure in complex with Smoothend (PDB: 6OT0) (Qi et al., 2019) and scFv16 from the X-ray crystal structure in complex with heterotrimeric G protein (PDB: 6CRK) (Maeda et al., 2018). Models were fit into EM maps using UCSF Chimera (Pettersen et al., 2004), and then rigid-body-fit using PHENIX (Liebschner et al., 2019), followed by iterative rounds of model rebuilding in Coot (Casañal et al., 2020) and ISOLDE (Croll, 2018), and real-space refinement in PHENIX. Restrains for all ligands were generated from the GRADE server (https://grade.globalphasing.org). Model validation was performed with MolProbity (Williams et al., 2018) and the wwPDB validation server (Berman et al., 2003). Figures were generated using UCSF Chimera (Pettersen et al., 2004), Chimera X (Pettersen et al., 2021), and PyMOL (Schrödinger).

### Cryo-EM 3D variability analysis

3D variability analysis (3DVAR) was performed to access and visualize the dynamics within the cryo-EM datasets of the M_4_ mAChR complexes, as previously described using cryoSPARC (Punjani and Fleet, 2021). The polished particle stacks were imported into cryoSPARC, followed by 2D classification and 3D refinement using the respective low-pass-filtered RELION consensus maps as an initial model. 3DVA was analyzed in three components with 20 volume frames of data per component of motion. Output files were visualized using UCSF Chimera (Pettersen et al., 2004).

### Gaussian accelerated molecular dynamics (GaMD)

GaMD enhances the conformational sampling of biomolecules by adding a harmonic boost potential to reduce the system energy barriers (Miao et al., 2015). When the system potential $V\left(\overset{⃑}{r}\right)$ is lower than a reference energy E, the modified potential $V^{}\left(\overset{⃑}{r}\right)$ of the system is calculated as

$$V^{}\left(\overset{⃑}{r}\right)=V\left(\overset{⃑}{r}\right)+∆V\left(\overset{⃑}{r}\right)$$

(1) $$\Delta V\left(\vec{r}\right)=\left\{ \begin{array}{l} \frac{1}{2}k{\left(E-V\left(\vec{r}\right)\right)}^{2},V\left(\vec{r}\right)<E\\ 0,\;\;\;V\left(\vec{r}\right)\geq E, \end{array}\right.$$

where k is the harmonic force constant. The two adjustable parameters E and k are automatically determined on three enhanced sampling principles. First, for any two arbitrary potential values $v_{1}\left(\overset{⃑}{r}\right)$ and $v_{2}\left(\overset{⃑}{r}\right)$ found on the original energy surface, if $V_{1}\left(\vec{r}\right)<V_{2}\left(\vec{r}\right)$ , ∆V should be a monotonic function that does not change the relative order of the biased potential values; that is, $V_{1}^{}\left(\vec{r}\right)<V_{2}^{}\left(\vec{r}\right)$ . Second, if $V_{1}\left(\vec{r}\right)<V_{2}\left(\vec{r}\right)$ , the potential difference observed on the smoothened energy surface should be smaller than that of the original; i.e., $V_{2}^{}\left(\vec{r}\right)-V_{1}^{}\left(\vec{r}\right)<V_{2}\left(\vec{r}\right)-V_{1}\left(\vec{r}\right)$ . By combining the first two criteria and plugging in the formula of $V^{}\left(\overset{⃑}{r}\right)$ and ∆V, we obtain

, (2) $$V_{max}\leq E\leq V_{min}+\frac{1}{k}$$

where $V_{min}$ and $V_{max}$ are the system minimum and maximum potential energies. To ensure that Equation 2 is valid, *k* has to satisfy $k\leq 1/\left(V_{max}-V_{min}\right)$ . Let us define $k=k_{0}∙1/\left(V_{max}-V_{min}\right)$ , then $0k_{0}\leq 1$. Third, the standard deviation (SD) of ∆V needs to be small enough (i.e. narrow distribution) to ensure accurate reweighting using cumulant expansion to the second order: $\sigma_{\Delta V}=k\left(E-V_{avg}\right)\sigma_{V}\leq \sigma_{0}$ , where $V_{avg}$ and $\sigma_{V}$ are the average and SD of ∆V with $\sigma_{0}$ as a user-specified upper limit (e.g. ${10k}_{B}T$) for accurate reweighting. When E is set to the lower bound $E=V_{max}$ according to Equation 2, $k_{0}$ can be calculated as

, (3) $$k_{0}=min\left(1.0,k_{0}^{`}\right)=min\left(1.0,\frac{\sigma_{0}}{\sigma_{V}}∙\frac{V_{max}-V_{min}}{V_{max}-V_{avg}}\right)$$

Alternatively, when the threshold energy E is set to its upper bound $E=V_{min}+1/k$, $k_{0}$ is set to

, (4) $$k_{0}=k_{0}^{``}\equiv \left(1-\frac{\sigma_{0}}{\sigma_{V}}\right)∙\frac{V_{max}-V_{min}}{V_{avg}-V_{min}}$$

If $k_{0}^{``}$ is calculated between 0 and 1. Otherwise, $k_{0}$ is calculated using Equation 3.

### Energetic reweighting of GaMD simulations

For energetic reweighting of GaMD simulations to calculate potential of mean force (PMF), the probability distribution along a reaction coordinate is written as $p^{}\left(A\right)$ . Given the boost potential $∆V\left(r\right)$ of each frame, $p^{}\left(A\right)$ can be reweighted to recover the canonical ensemble distribution $p\left(A\right)$ , as

, (5) $$p\left(A_{j}\right)=p^{}\left(A_{j}\right)\frac{{\left\langle e^{\beta ∆V\left(r\right)}\right\rangle }_{j}}{\sum_{i=1}^{M}{\left\langle {p^{}\left(A_{i}\right)e}^{\beta ∆V\left(r\right)}\right\rangle }_{i}},j=1,\ldots ,M$$

where *M* is the number of bins, $\beta =k_{B}T$, and ${\left\langle e^{\beta ∆V\left(r\right)}\right\rangle }_{j}$ is the ensemble-averaged Boltzmann factor of $∆V\left(r\right)$ for simulation frames found in the *j*th bin. The ensemble-averaged reweighting factor can be approximated using cumulant expansion:

, (6) $$\left\langle e^{\beta ∆V\left(r\right)}\right\rangle =exp\left\{\sum_{k=1}^{\infty }\frac{\beta^{k}}{k!}C_{k}\right\}$$

where the first two cumulants are given by

(7) $$\begin{array}{c} C_{1}=\left\langle ∆V\right\rangle ,\\ C_{2}=\left\langle ∆V^{2}\right\rangle -{\left\langle ∆V\right\rangle }^{2}=\sigma_{v}^{2}. \end{array}$$

The boost potential obtained from GaMD simulations usually follows near-Gaussian distribution (Miao and McCammon, 2017). Cumulant expansion to the second order thus provides a good approximation for computing the reweighting factor (Miao et al., 2015; Miao et al., 2014). The reweighted free energy $F\left(A\right)={-k}_{B}Tlnp\left(A\right)$ is calculated as

, (8) $$F\left(A\right)=F^{}\left(A\right)-\sum_{k=1}^{2}\frac{\beta^{k}}{k!}C_{k}+F_{c}$$

where $F^{}\left(A\right)={-k}_{B}Tlnp^{}\left(A\right)$ is the modified free energy obtained from GaMD simulation and $F_{c}$ is a constant.

### System setup

The M_4_R-ACh-G_i1_, M_4_R-Ipx-G_i1_, M_4_R-Ipx-G_i1_-VU154, and M_4_R-Ipx-G_i1_-LY298 cryo-EM structures were used for setting up simulation systems. The scFv16 in the cryo-EM structures was omitted in all simulations. The initial structures of single mutant D432E and T433R mutant of M_4_R-Ipx-G_i1_-VU154 were obtained by mutating the corresponding residues in the M_4_R-Ipx-G_i1_-VU154 cryo-EM structure. The initial structures of M_4_R-ACh-G_i1_-VU154 and M_4_R-ACh-G_i1_-LY298 were obtained from M_4_R-Ipx-G_i1_-VU154 and M_4_R-Ipx-G_i1_-LY298 cryo-EM structures by replacing Ipx with ACh through alignment of receptors to the M4R-ACh-G_i1_ cryo-EM structure. The initial structures of M_4_R-G_i1_-VU154 and M_4_R-G_i1_-LY298 were obtained by removing the corresponding Ipx agonist from the M_4_R-Ipx-G_i1_-VU154 and M_4_R-Ipx-G_i1_-LY298 cryo-EM structures. The initial structures of M_4_R-VU154 and M_4_R-LY298 were obtained by removing the corresponding Ipx agonist and G_i1_ protein from the M_4_R-Ipx-G_i1_-VU154 and M_4_R-Ipx-G_i1_-LY298 cryo-EM structures. According to previous findings, intracellular loop (ICL) 3 is highly flexible and removal of ICL3 does not appear to affect GPCR function (Dror et al., 2015; Dror et al., 2011). The ICL3 was thus omitted as in the current GaMD simulations. Similar to a previous study, helical domains of the G_i1_ protein missing in the cryo-EM structures were not included in the simulation models. This was based on earlier simulation of the β_2_AR-G_s_ complex, which showed that the helical domain fluctuated substantially (Dror et al., 2015). All chain termini were capped with neutral groups (acetyl and methylamide). All the disulfide bonds in the complexes (i.e. Cys108^3.25^-Cys185^45x50^ and Cys426^ECL3^-Cys429^ECL3^ in the M4R) that were resolved in the cryo-EM structures were maintained in the simulations. Using the *psfgen* plugin in VMD (Humphrey et al., 1996), missing atoms in protein residues were added and all protein residues were set to the standard CHARMM protonation states at neutral pH. For each of the complex systems, the receptor was inserted into a palmitoyl-oleoyl-phosphatidyl-choline (POPC) bilayer with all overlapping lipid molecules removed using the membrane plugin in VMD. The system charges were then neutralized at 0.15 M NaCl using the *solvate* plugin in VMD (Humphrey et al., 1996). The simulation systems were summarized in Table 3.

### Simulation protocol

The CHARMM36M parameter set (Huang et al., 2017; Klauda et al., 2010; Vanommeslaeghe and MacKerell, 2015) was used for the M_4_ mAChRs, G_i1_ proteins, and POPC lipids. Force field parameters of agonists ACh and Ipx, PAMs LY298 and VU154 were obtained from the CHARMM ParamChem web server (Vanommeslaeghe et al., 2012b; Vanommeslaeghe and MacKerell, 2012a). Force field parameters with high penalty were optimized with FFParm (Kumar et al., 2020). GaMD simulations of these systems followed a similar protocol used in previous studies of GPCRs (Draper-Joyce et al., 2021; Miao and McCammon, 2018; Miao and McCammon, 2016). For each of the complex systems, initial energy minimization, thermalization, and 20 ns cMD equilibration were performed using NAMD2.12 (Phillips et al., 2005). A cutoff distance of 12 Å was used for the van der Waals and short-range electrostatic interactions and the long-range electrostatic interactions were computed with the particle-mesh Ewald summation method (Darden et al., 1993). A 2-fs integration time step was used for all MD simulations, and a multiple-time-stepping algorithm was used with bonded and short-range non-bonded interactions computed every time step and long-range electrostatic interactions every two-time steps. The SHAKE algorithm (Ryckaert et al., 1977) was applied to all hydrogen-containing bonds. The NAMD simulation started with equilibration of the lipid tails. With all other atoms fixed, the lipid tails were energy minimized for 1000 steps using the conjugate gradient algorithm and melted with a constant number, volume, and temperature (NVT) run for 0.5 ns at 310 K. The 12 systems were further equilibrated using a constant number, pressure, and temperature (NPT) run at 1 atm and 310 K for 10 ns with 5 kcal/(mol. Å^2^) harmonic position restraints applied to the protein and ligand atoms. Final equilibration of each system was performed using a NPT run at 1 atm pressure and 310 K for 0.5 ns with all atoms unrestrained. After energy minimization and system equilibration, conventional MD simulations were performed on each system for 20 ns at 1 atm pressure and 310 K with a constant ratio constraint applied on the lipid bilayer in the X-Y plane.

With the NAMD output structure, along with the system topology and CHARMM36M force field files, the *ParmEd* tool in the AMBER package was used to convert the simulation files into the AMBER format. The GaMD module implemented in the GPU version of AMBER20 (Case et al. 2020) was then applied to perform the GaMD simulation. GaMD simulations of systems with G_i1_ protein (M_4_R-ACh-G_i1_, M_4_R-Ipx-G_i1_, M_4_R-Ipx-G_i1_-VU154, M_4_R-Ipx-G_i1_-LY298, M_4_R-ACh-G_i1_-VU154, M_4_R-ACh-G_i1_-LY298, single mutant D432E and T433R mutants of M_4_R-Ipx-G_i1_-VU154) included an 8-ns short cMD simulation used to collect the potential statistics for calculating GaMD acceleration parameters, a 48-ns equilibration after adding the boost potential, and finally three independent 500-ns GaMD production simulations with randomized initial atomic velocities. The average and SD of the system potential energies were calculated every 800,000 steps (1.6 ns). GaMD simulations of M_4_R-VU154 and M_4_R-LY298 included a 2.4-ns short cMD simulation used to collect the potential statistics for calculating GaMD acceleration parameters, a 48-ns equilibration after adding the boost potential, and finally three independent 1000-ns GaMD production simulations with randomized initial atomic velocities. The average and SD of the system potential energies were calculated every 240,000 steps (0.48 ns). All GaMD simulations were run at the ‘dual-boost’ level by setting the reference energy to the lower bound. One boost potential is applied to the dihedral energetic term and the other to the total potential energetic term. The upper limit of the boost potential SD, σ_0_ was set to 6.0 kcal/mol for both the dihedral and the total potential energetic terms. Similar temperature and pressure parameters were used as in the NAMD simulations.

### Simulation analysis

CPPTRAJ (Roe and Cheatham, 2013) and VMD (Humphrey et al., 1996) were used to analyze the GaMD simulations. The RMSDs of the agonist ACh and Ipx, PAM VU154 and LY298 relative to the simulation starting structures, the interactions between receptor and agonists/PAMs, distances between the receptor TM3 and TM6 intracellular ends were selected as reaction coordinates. Particularly, distances were calculated between the Cα atoms of residues Arg^3.50^ and Thr^6.30^, N atom of residue N117^3.37^ and carbon atom (C5) in the acetyl group of ACh or oxygen atom (O09) in the ether bond of Ipx, NE1 atom of residue W164^4.67^ and carbon atom (C5) in the acetyl group of ACh or oxygen atom (O09) in the ether bond of Ipx, indole ring of residue W413^6.48^ and acetyl group of ACh or heterocyclic isoazoline group of Ipx, OH atom of residue Y89^2.61^ and oxygen atom in the amide group of VU154/LY298, benzene ring of residue F186^45.51^ and aromatic core of the PAMs VU154/LY298, OH atom of residue Y439^7.39^ and nitrogen atoms in the amine group of the PAMs VU154/LY298, CD atom of residue Q184^45.49^ and nitrogen atom in the amide group of VU154/LY298, CG atom of residue N423^6.58^ and chlorine atom in PAM LY298, OH atom of residue Y92^2.64^ and nitrogen atom in the amide group of VU154, OG1 atom of residue T433^7.33^ and sulfur atom in the trifluoromethylsulfonyl group of VU154. In addition, the χ_2_ angle of residue W413^6.48^ and W435^7.35^ were calculated. Time courses of these reaction coordinates obtained from the GaMD simulation were plotted in the respective figures. The PyReweighting (Miao et al., 2014) toolkit was applied to reweight GaMD simulations to recover the original free energy or PMF profiles of the simulation systems. PMF profiles were computed using the combined trajectories from all the three independent 500 ns GaMD simulations for each system. A bin size of 1.0 Å was used for RMSD. The cutoff was set to 500 frames for 2D PMF calculations. The 2D PMF profiles were obtained for wildtype M_4_R-Ipx-G_i1_-LY298, M_4_R-Ipx-G_i1_-VU154, and the D432E and T433R single mutants of the M_4_R-Ipx-G_i1_-VU154 system regarding the RMSDs of the agonist Ipx and the RMSDs of the PAMs relative to the cryo-EM conformation.

### Data analysis

All pharmacological data was fit using GraphPad Prism 9.2.0. Saturation binding experiments to determine B_max_ and pK_d_ values were determined as previously described (Leach et al., 2011; Nawaratne et al., 2010; Thal et al., 2016). Detailed equations and analysis details can be found in Appendix 1. Interaction inhibition binding curves between [^3^H]-NMS, agonists (ACh or Ipx), and PAMs (LY298 or VU154) were analyzed using the allosteric ternary complex model to calculate binding affinity values for each ligand (pK_A_ – for ACh/Ipx and pK_B_ for LY298/VU154) and the degree of binding modulation between agonist and PAM (log α) (Christopoulos and Kenakin, 2002). The pK_B_ values for LY298 and VU154 were determined from global fits of the ACh and Ipx curves to generate one pK_B_ value per ligand (Ehlert, 1988; Leach et al., 2011; Nawaratne et al., 2010; Thal et al., 2016). All pERK1/2 and TruPath assays were analyzed using the operational model allosterism and agonism to determine values of orthosteric (τ_A_) or allosteric efficacy (τ_B_) and the functional modulation (log αβ) between the agonists and PAMs (Leach et al., 2011; Nawaratne et al., 2010). Binding affinities of the agonists and the PAMs were fixed to values determined from equilibrium binding assays. The τ_B_ values for LY298 and VU154 were determined from global fits of the ACh and Ipx curves (when possible) to generate one value per ligand. For comparison between WT human M_4_ mAChR and other M_4_ mAChR constructs, the log τ values were corrected (denoted log τ_C_) by normalizing to B_max_ values from saturation binding experiments (Leach et al., 2011; Nawaratne et al., 2010; Thal et al., 2016). All affinity, potency, and cooperativity values were estimated as logarithms, and statistical analysis between WT and mutant M_4_ mAChR was determined by one-way ANOVA using a Dunnett’s post-hoc test with a value of p<0.05 considered as significant in this study.

## Data Availability

All data generated or analysed during this study are included in the manuscript. Structural data has been deposited in the Protein Data Bank (PDB) and Electron Microscopy Data Bank (EMDB) under the following codes:(1) M4R-Gi1-Ipx PDB: 7TRK and EMD-26099 (2) M4R-Gi1-Ipx-LY298 PDB: 7TRP and EMD-26100 (3) M4R-Gi1-Ipx-VU154 PDB: 7TRQ and EMD-26101 (4) M4R-Gi1-ACh PDB: 7TRS and EMD-26102. The following datasets were generated: VuckovicZ MobbsJI BelousoffMJ GlukhovaA SextonPM DanevR ThalDM 2023Human M4 muscarinic acetylcholine receptor complex with Gi1 and the agonist iperoxoRCSB Protein Data Bank7TRK VuckovicZ MobbsJI BelousoffMJ GlukhovaA SextonPM DanevR ThalDM 2023Human M4 muscarinic acetylcholine receptor complex with Gi1 and the agonist iperoxo and positive allosteric modulator LY2033298RCSB Protein Data Bank7TRP VuckovicZ MobbsJI BelousoffMJ GlukhovaA SextonPM DanevR ThalDM 2023Human M4 muscarinic acetylcholine receptor complex with Gi1 and the agonist iperoxo and positive allosteric modulator VU0467154RCSB Protein Data Bank7TRQ VuckovicZ MobbsJI BelousoffMJ GlukhovaA SextonPM DanevR ThalDM 2023Human M4 muscarinic acetylcholine receptor complex with Gi1 and the endogenous agonist acetylcholineRCSB Protein Data Bank7TRS VuckovicZ MobbsJI BelousoffMJ GlukhovaA SextonPM DanevR ThalDM 2023Human M4 muscarinic acetylcholine receptor complex with Gi1 and the agonist iperoxoElectron Microscopy Data BankEMD-26099 VuckovicZ MobbsJI BelousoffMJ GlukhovaA SextonPM DanevR ThalDM 2023Human M4 muscarinic acetylcholine receptor complex with Gi1 and the agonist iperoxo and positive allosteric modulator LY2033298Electron Microscopy Data BankEMD-26100 VuckovicZ MobbsJI BelousoffMJ GlukhovaA SextonPM DanevR ThanlDM 2023Human M4 muscarinic acetylcholine receptor complex with Gi1 and the agonist iperoxo and positive allosteric modulator VU0467154Electron Microscopy Data BankEMD-26101 VuckovicZ MobbsJI BelousoffMJ GlukhovaA SextonPM DanevR ThalDM 2023Human M4 muscarinic acetylcholine receptor complex with Gi1 and the endogenous agonist acetylcholineElectron Microscopy Data BankEMD-26102

## Additional files

MDAR checklist

## Appendix 1

### Data analysis

#### Pharmacological parameters related to ligand binding

Interaction radioligand binding data were analyzed according to the following adapted form of an allosteric ternary complex model that accounts for the interaction of two orthosteric ligands and one allosteric ligand on a receptor (Christopoulos and Kenakin, 2002; Leach et al., 2007; Leach et al., 2011):

$$Y=\frac{B_{max}\cdot \left[A\right]}{\left[A\right]+\left[\left(K_{A}\cdot K_{B})/(\alpha_{A}[B]+K_{B}\right)\right]\cdot \left\{1+([I]/K_{I})+([B]/K_{B})+[(\alpha_{I}[I][B]/K_{I}K_{B}])\right\}}$$

- [A], [B], and [I] represent the concentrations of the radioligand ([^3^H]-NMS), allosteric ligand, and orthosteric inhibitor, respectively.
- *K_A_*, *K_B_*, and *K_I_* represent their respective equilibrium dissociation constants. The value *K_A_* was fixed to the value determined from saturation binding experiments.
- B_max_ is the total number of receptors.
- *α_A_* and *α_I_* represent the affinity cooperativity values between the allosteric ligand and the radioligand or orthosteric inhibitor, respectively. Values greater than 1 indicate positive cooperativity, values <1 (but >0) indicate negative cooperativity, and values of unity indicate neutral cooperativity.
- All potency, affinity, and cooperativity parameters were estimated as logarithms.

Agonist binding affinity with a PAM bound:

$$Y=pK_{I}+log\;\alpha_{I}$$

$$\Delta Y=((\Delta pK_{I}{)}^{2}+(\Delta log\;\alpha_{I}{)}^{2}{)}^{1/2}$$

PAM binding affinity for the agonist-bound state:

$$Y=pK_{B}+log\;\alpha_{I}$$

$$\Delta Y=((\Delta pK_{B}{)}^{2}+(\Delta log\;\alpha_{I}{)}^{2}{)}^{1/2}$$

Global analysis for sharing the pK_B_ value between interaction experiments using two different agonists:

Define:

Ag1=agonist 1 (e.g. ACh)

Ag2=agonist 2 (e.g. Ipx)

alpha = *α_A_*

betta = *α_I_*

Data for agonist 1 in columns <A:F>

Data for agonist 2 in columns <G:L>

Prism equation:

$$KA=10^{\wedge }LogKA$$

$$KB=10^{\wedge }LogKB$$

$$I=10^{\wedge }X$$

$$A=10^{\wedge }LogHotA$$

$$KIAg1=10^{\wedge }LogKIAg1\;(Ki\;for\;agonist\;1)$$

$$KIAg2=10^{\wedge }LogKIAg2\;(Ki\;for\;agonist\;2)$$

$$alphaAg1=10^{\wedge }LogalphaAg1$$

$$bettaAg1=10^{\wedge }LogbettaAg1$$

$$alphaAg2=10^{\wedge }LogalphaAg2$$

$$bettaAg2=10^{\wedge }LogbettaAg2$$

$$Part1Ag1=(KA^{*}KB)/(alphaAg1^{*}B+KB)$$

$$Part2Ag1=1+I/KIAg1+B/KB+(bettaAg1^{*}I^{*}B)/(KIAg1^{*}KB)$$

$$KAppAg1=Part1Ag1^{*}Part2Ag1$$

$$Part1Ag2=(KA^{*}KB)/(alphaAg2^{*}B+KB)$$

$$Part2Ag2=1+I/KIAg2+B/KB+(bettaAg2^{*}I^{*}B)/(KIAg2^{*}KB)$$

$$KAppAg2=Part1Ag2^{*}Part2Ag2$$

$$<A:F>Y=(BmaxAg1^{*}A)/(A+KAppAg1)$$

$$<G:L>Y=(BmaxAg1^{*}A)/(A+KAppAg2)$$

#### Pharmacological parameters related to function

To determine efficacy values ($\tau_{A}$) of ACh and Ipx in TruPath and pERK1/2 assays, data were directly fit to the operational model of agonism (Black and Leff, 1983):

$$Y=Basal+\frac{E_{m}-Basal}{1+\frac{K_{A}+\left[A\right]}{\tau_{A}\times \left[A\right]}}$$

where $E_{m}$ is the maximal response of the system, Basal is the basal level of response in the absence of agonist, [A] is the concentration of agonist, K_A_ denotes the equilibrium dissociation constant of the agonist (A), and $\tau_{A}$ is the index of the coupling efficiency/efficacy of the agonist. Values of K_A_ were constrained to the corresponding K_I_ values from interaction radioligand binding experiments.

The determination of $\tau_{B}$ and αβ values for LY298 and VU154 in TruPath and pERK1/2 assays data was directly fit to the following operational model of allosterism and agonism (Leach et al., 2007):

$$Y=Basal+\frac{{\left(E_{m}-Basal\right)\left(\left[A\right]K_{B}+\alpha \beta \left[A\right]\left[B\right]+\tau_{B}\left[A\right]\left[B\right]{EC}_{50}\right)}^{n}}{{\left(\left[A\right]K_{B}+\alpha \beta \left[A\right]\left[B\right]+\tau_{B}\left[A\right]\left[B\right]{EC}_{50}\right)}^{n}+{{EC}_{50}}^{n}{\left(K_{B}+\left[B\right]\right)}^{n}}$$

where $E_{m}$ is the maximal response of the system, Basal is the basal level of response in the absence of agonist, [A] and [B] are the concentrations of agonist and the PAM, respectively. K_B_ denotes the equilibrium dissociation constant of the PAM. $\tau_{B}$ is the index of the coupling efficiency/efficacy of the PAM. α denotes the binding cooperativity between the agonist and PAM, whereas β denotes a scaling factor that quantifies the allosteric effect of the PAM on the orthosteric ligand efficacy. This model assumes that the agonists (A) are full agonists. n is the transducer slope that describes the stimulus–response coupling of the ligand–receptor to the signaling pathway and was constrained to 1. The allosteric binding affinity ($K_{B}$) was constrained to the value determined from radioligand binding experiments. All potency, affinity, efficacy, and cooperativity parameters were estimated as logarithms.

Transduction coupling coefficients were calculated as follows Kenakin et al., 2012:

$$Y=log\frac{\tau_{A}}{K_{A}}=log\tau_{A}-logK_{A}$$

$$\Delta Y=((\Delta log\tau_{A}{)}^{2}+(\Delta K_{A}{)}^{2}{)}^{1/2}$$

Efficacy modulation (β) was calculated as follows:

Y=logβ=logαβ−logα

$$\Delta Y=((\Delta log\;\alpha \beta {)}^{2}+(\Delta log\;\alpha {)}^{2}{)}^{1/2}$$

Global analysis for sharing $\tau_{B}$ between interaction functional experiments using two different agonists:

Define:

Ag1=agonist 1 (e.g. ACh)

Ag2=agonist 2 (e.g. Ipx)

ab=αβ

Data for agonist 1 in columns <A:F>

Data for agonist 2 in columns <G:L>

Prism equation:

$$KB=10^{\wedge }LogKB$$

$$tauB=10^{\wedge }LogtauB$$

$$A=10^{\wedge }X$$

B=ConcMod

$$EC50Ag1=10^{\wedge }LogEC50Ag1$$

$$EC50Ag2=10^{\wedge }LogEC50Ag2$$

$$abAg1=10^{\wedge }LogAlphaBetaAg1$$

$$abAg2=10^{\wedge }LogAlphaBetaAg2$$

$$Part1Ag1=(A^{*}(KB+abAg1^{*}B)+tauB^{*}B^{*}EC50Ag1{)}^{\wedge }n$$

$$Part1Ag2=(A^{*}(KB+abAg2^{*}B)+tauB^{*}B^{*}EC50Ag2{)}^{\wedge }n$$

$$Part2Ag1=(EC50Ag1^{\wedge }n{)}^{*}((KB+B{)}^{\wedge }n)$$

$$Part2Ag2=(EC50Ag2^{\wedge }n{)}^{*}((KB+B{)}^{\wedge }n)$$

SpanAg1=EmAg1‐BasalAg1

SpanAg2=EmAg2‐BasalAg2

$$<A:F>Y=BasalAg1+(SpanAg1^{*}Part1Ag1)/(Part1Ag1+Part2Ag1)$$

$$<G:L>Y=BasalAg2+(SpanAg2^{*}Part1Ag2)/(Part1Ag2+Part2Ag2)$$

Correcting efficacy $\tau_{X}$ values using receptor expression (Leach et al., 2011; Nawaratne et al., 2010):

B_max–WT_ = maximal number of receptors for the WT receptor

B_max–X_ = maximal number of receptors for the receptor construct X

$\tau_{WT}$ = efficacy at the WT receptor

$\tau_{X}$ = efficacy at receptor X

$$log\tau_{X-corr}=log\tau_{X}-(log\;B_{max-X}-log\;B_{max-WT})$$

$$\Delta log\tau_{X-corr}={\left({\left(\Delta log\tau_{X}\right)}^{2}+{\left(0.432\times \left(\frac{\Delta B_{max-X}}{B_{max-X}}\right)\right)}^{2}+{\left(0.432\times \left(\frac{\Delta B_{max-WT}}{B_{max-WT}}\right)\right)}^{2}\right)}^{1/2}$$

**Appendix 1—key resources table:**

Reagent type (species) or resource	Designation	Source or reference	Identifiers	Additional information
Antibody	Anti-FLAG M1 (mouse polyclonal IgG2a)	Gift from Prof. Brian Kobilka (PMID::17962520)		Antibody was used to make anti-FLAG mAb resin that was used for the purification of FLAG-tagged M4 mAChR
Strain, strain background (*Escherichia coli*)	DH5α	New England Biolabs	C2987H	
Strain, strain background (*E. coli*)	DH10bac	Thermo Fisher Scientific	10361012	
Cell line (*Spodoptera frugiperda*)	Sf9	Expression Systems	94-001S	
Cell line (*Trichoplusia ni*)	Tni	Expression Systems	94-002S	
Cell line (Chinese hamster ovary)	Flp-In CHO human M4 mAChR WT	PMID:26958838		
Cell line (Chinese hamster ovary)	CHO K1 mouse M4 mAChR WT	PMID:21198541		
Cell line (Chinese hamster ovary)	Flp-In CHO human M4 mAChR D432E	This study		pEF5-FRT-V5-DEST plasmid
Cell line (Chinese hamster ovary)	Flp-In CHO human M4 mAChR T433R	This study		pEF5-FRT-V5-DEST plasmid
Cell line (Chinese hamster ovary)	Flp-In CHO human M4 mAChR V91L, D432E, T433R	This study		pEF5-FRT-V5-DEST plasmid
Cell line (Chinese hamster ovary)	Flp-In CHO human M4 mAChR Y89A	PMID:26958838		
Cell line (Chinese hamster ovary)	Flp-In CHO human M4 mAChR Q184A	PMID:26958838		
Cell line (Chinese hamster ovary)	Flp-In CHO human M4 mAChR F186A	PMID:20406819		
Cell line (Chinese hamster ovary)	Flp-In CHO human M4 mAChR W435A	PMID:26958838		
Cell line (Chinese hamster ovary)	Flp-In CHO human M4 mAChR W439A	PMID:20406819		
Cell line (Chinese hamster ovary)	Flp-In CHO human M4 mAChR S85A	PMID:26958838		
Cell line (Chinese hamster ovary)	Flp-In CHO human M4 mAChR Y89A	PMID:26958838		
Cell line (Chinese hamster ovary)	Flp-In CHO human M4 mAChR Y92A	PMID:26958838		
Cell line (Chinese hamster ovary)	Flp-In CHO human M4 mAChR I93T, I94V, K95I	PMID:20406819		
Cell line (Chinese hamster ovary)	Flp-In CHO human M4 mAChR I93T	PMID:20406819		
Cell line (Chinese hamster ovary)	Flp-In CHO human M4 mAChR I94V	PMID:20406819		
Cell line (Chinese hamster ovary)	Flp-In CHO human M4 mAChR K95I	PMID:20406819		
Cell line (Chinese hamster ovary)	Flp-In CHO human M4 mAChR Y97A	PMID:26958838		
Cell line (Chinese hamster ovary)	Flp-In CHO human M4 mAChR W98A	PMID:26958838		
Cell line (Chinese hamster ovary)	Flp-In CHO human M4 mAChR G101A	PMID:26958838		
Cell line (Chinese hamster ovary)	Flp-In CHO human M4 mAChR D106A	PMID:21300722		
Cell line (Chinese hamster ovary)	Flp-In CHO human M4 mAChR W108A	PMID:21300722		
Cell line (Chinese hamster ovary)	Flp-In CHO human M4 mAChR L109A	PMID:21300722		
Cell line (Chinese hamster ovary)	Flp-In CHO human M4 mAChR D112E	PMID:21300722		
Cell line (Chinese hamster ovary)	Flp-In CHO human M4 mAChR D112N	PMID:21300722		
Cell line (Chinese hamster ovary)	Flp-In CHO human M4 mAChR S116A	PMID:21300722		
Cell line (Chinese hamster ovary)	Flp-In CHO human M4 mAChR N117A	PMID:21300722		
Cell line (Chinese hamster ovary)	Flp-In CHO human M4 mAChR V120A	PMID:21300722		
Cell line (Chinese hamster ovary)	Flp-In CHO human M4 mAChR D129E	PMID:21300722		
Cell line (Chinese hamster ovary)	Flp-In CHO human M4 mAChR D129N	PMID:21300722		
Cell line (Chinese hamster ovary)	Flp-In CHO human M4 mAChR W164A	PMID:26958838		
Cell line (Chinese hamster ovary)	Flp-In CHO human M4 mAChR F170A	PMID:26958838		
Cell line (Chinese hamster ovary)	Flp-In CHO human M4 mAChR W171A	PMID:26958838		
Cell line (Chinese hamster ovary)	Flp-In CHO human M4 mAChR Q172A	PMID:26958838		
Cell line (Chinese hamster ovary)	Flp-In CHO human M4 mAChR F173A	PMID:26958838		
Cell line (Chinese hamster ovary)	Flp-In CHO human M4 mAChR Q184A	PMID:26958838		
Cell line (Chinese hamster ovary)	Flp-In CHO human M4 mAChR F186A	PMID:20406819		
Cell line (Chinese hamster ovary)	Flp-In CHO human M4 mAChR I187A	PMID:26958838		
Cell line (Chinese hamster ovary)	Flp-In CHO human M4 mAChR Q188A	PMID:26958838		
Cell line (Chinese hamster ovary)	Flp-In CHO human M4 mAChR F189A	PMID:26958838		
Cell line (Chinese hamster ovary)	Flp-In CHO human M4 mAChR L190A	PMID:26958838		
Cell line (Chinese hamster ovary)	Flp-In CHO human M4 mAChR W413A	PMID:26958838		
Cell line (Chinese hamster ovary)	Flp-In CHO human M4 mAChR Y416A	PMID:26958838		
Cell line (Chinese hamster ovary)	Flp-In CHO human M4 mAChR N423A	PMID:26958838		
Cell line (Chinese hamster ovary)	Flp-In CHO human M4 mAChR Q427A	PMID:26958838		
Cell line (Chinese hamster ovary)	Flp-In CHO human M4 mAChR S428P	PMID:20406819		
Cell line (Chinese hamster ovary)	Flp-In CHO human M4 mAChR D432N	PMID:20406819		
Cell line (Chinese hamster ovary)	Flp-In CHO human M4 mAChR W435A	PMID:26958838		
Cell line (Chinese hamster ovary)	Flp-In CHO human M4 mAChR Y439A	PMID:20406819		
Cell line (Chinese hamster ovary)	Flp-In CHO human M4 mAChR Y440A	PMID:26958838		
Cell line (Chinese hamster ovary)	Flp-In CHO human M4 mAChR C442A	PMID:20406819		
Cell line (Chinese hamster ovary)	Flp-In CHO human M4 mAChR Y443A	PMID:20406819		
Cell line (Chinese hamster ovary)	Flp-In CHO cell line	Thermo Fisher Scientific	R75807	
Cell line (*Homo sapiens*)	293A cell line	Thermo Fisher Scientific	R70507	
Recombinant DNA reagent	Human FLAG-M4∆i3-His	This study		pVL1392 vector
Recombinant DNA reagent	Human Gαi1 dominant negative mutant	PMID:32193322		pFastBac vector
Recombinant DNA reagent	Human Gβ1γ2	Gift from Prof. Brian Kobilka (PMID:24256733)		pVL1392 vector
Recombinant DNA reagent	scFv16	PMID:30213947		pFastBac vector
Recombinant DNA reagent	pcDNA5/FRT/TO-GAlphai1-RLuc8	Gift from Prof. Bryan Roth (PMID:32367019)		TRUPATH assay
Recombinant DNA reagent	pcDNA3.1-Beta3	Gift from Prof. Bryan Roth (PMID:32367019)		TRUPATH assay
Recombinant DNA reagent	pcDNA3.1-GGamma9-GFP2	Gift from Prof. Bryan Roth (PMID:32367019)		TRUPATH assay
Chemical compound, drug	Acetylcholine	Sigma-Aldrich		
Chemical compound, drug	Iperoxo	Sigma-Aldrich		
Chemical compound, drug	LY2033298	Sigma-Aldrich		
Chemical compound, drug	VU0467154	Gift from Prof. Craig Lindsley (PMID:25137629)		PMID:25137629
Chemical compound, drug	Prolume Purple	Nanolight Technology		
Chemical compound, drug	[^3^H]-NMS	PerkinElmer		
Chemical compound, drug	Polyethylenimine (PEI)	Sigma-Aldrich		
Chemical compound, drug	Atropine	Sigma-Aldrich		
Commercial assay or kit	AlphaScreen SureFire pERK 1/2 (Thr202/Tyr204) Assay Kits	PerkinElmer		
Software, algorithm	Prism 8.0	GraphPad		
Software, algorithm	PyMOL	Schrödinger		
Software, algorithm	GaMD	PMID:26300708		
Software, algorithm	AMBER20	https://ambermd.org		
Software, algorithm	CPPTRAJ	PMID:26300708		
Software, algorithm	PyReweighting	PMID:25061441		
Software, algorithm	Phenix suite	PMID:20124702		
Software, algorithm	Coot	PMID:31730249		
Software, algorithm	Chimera	PMID:15264254		
Software, algorithm	Chimera X	PMID:32881101		
Software, algorithm	cryoSPARC	PMID:28165473		
Software, algorithm	Relion 3.1	PMID:30412051		
Software, algorithm	Motioncor2	PMID:28250466		
Software, algorithm	GCTF	PMID:26592709		
Software, algorithm	crYOLO	PMID:31240256		
Software, algorithm	ISOLDE	PMID:29872003		
Software, algorithm	MolProbity	PMID:29067766		
Software, algorithm	DAQ score	PMID:35953671		
